# Satellite imagery-based monitoring of archaeological site damage in the Syrian civil war

**DOI:** 10.1371/journal.pone.0188589

**Published:** 2017-11-30

**Authors:** Jesse Casana, Elise Jakoby Laugier

**Affiliations:** 1 Department of Anthropology, Dartmouth College, Hanover, New Hampshire, United States of America; 2 Program in Ecology, Evolution, Ecosystems, and Society, Dartmouth College, Hanover, New Hampshire, United States of America; University at Buffalo - The State University of New York, UNITED STATES

## Abstract

Since the start of the Syrian civil war in 2011, the rich archaeological heritage of Syria and northern Iraq has faced severe threats, including looting, combat-related damage, and intentional demolition of monuments. However, the inaccessibility of the conflict zone to archaeologists or cultural heritage specialists has made it difficult to produce accurate damage assessments, impeding efforts to develop mitigation strategies and policies. This paper presents results of a project, undertaken in collaboration with the American Schools of Oriental Research (ASOR) and the US Department of State, to monitor damage to archaeological sites in Syria, northern Iraq, and southern Turkey using recent, high-resolution satellite imagery. Leveraging a large database of archaeological and heritage sites throughout the region, as well as access to continually updated satellite imagery from DigitalGlobe, this project has developed a flexible and efficient methodology to log observations of damage in a manner that facilitates spatial and temporal queries. With nearly 5000 sites carefully evaluated, analysis reveals unexpected patterns in the timing, severity, and location of damage, helping us to better understand the evolving cultural heritage crisis in Syria and Iraq. Results also offer a model for future remote sensing-based archaeological and heritage monitoring efforts in the Middle East and beyond.

## Introduction

With the outbreak of civil war in Syria during 2011, and the spread of military conflict and political upheaval throughout surrounding regions of the Middle East, the region’s rich cultural heritage faced unprecedented threats from looting, direct conflict-related damage, and ideologically-driven destruction of sites and monuments [1–5]. However, the inability of archaeologists or heritage officials to access most parts of the war-torn country has left us with scant verifiable information regarding the state of archaeological sites and monuments. Because it offers the ability to monitor damage across large regions from space, satellite imagery-based analysis of cultural heritage has become increasingly popular and numerous research teams around the world have begun to devote significant resources to remote sensing-based monitoring in conflict zones in Syria and beyond (e.g., [6–12]). With these efforts, a new set of questions have emerged, ranging from how best to implement a remote sensing-based monitoring strategy, what results of such investigations can and cannot tell us about the situation on the ground, and the degree to which such undertakings pose ethical problems in light of the unfolding human tragedy of which they are a part.

This paper presents results of a two-year effort undertaken by the authors, in collaboration with the American Schools of Oriental Research (ASOR) Cultural Heritage Initiatives (CHI). Unlike the direct observational and report-based data assembled by other parts of the CHI team (e.g., [4,5]), or individual analyses of key sites where damage is known to have already occurred such as at Nimrud [13] or Palmyra [14], this report details findings of a study to survey systematically thousands of sites across the region. Leveraging a large database of archaeological and heritage sites that includes not only locational data but also information on site morphology, periodization, and other attributes, our research seeks to catalogue both pre- and post-war damage resulting from looting, earthmoving, militarization, construction, and other activities. The development of this comprehensive assessment database enables us to query results in order to reveal spatial and temporal patterns in site damage that would otherwise not be apparent, addressing questions such as whether looting is more frequent in some areas as opposed to others, what types of sites are most at risk, and how these issues have evolved over the course of the conflict. This paper seeks to present both an overview of our findings regarding looting, military-related, and other forms of damage occurring in the context of the Syrian civil war, as well as a detailed discussion of the strengths and weaknesses of our methodology, which may serve as a model for future research in the Middle East and beyond.

## Background

While scholars recognized the possibility of identifying looting on high-resolution satellite imagery since it first became available, the high cost of these data largely prevented its use by researchers. Following the looting of the National Museum in Baghdad in 2003, wartime threats to cultural heritage destruction became a major issue of public concern, and in this context, a team from Stony Brook University secured funding to undertake satellite imagery-based analysis of looting in southern Iraq [15,16]. Through analysis of 1900 sites, the project revealed patterns in the types of sites that were most likely to be targeted and the timing of looting, as well as illustrating the potential of such an approach more broadly. However, this project required commercial imagery to be purchased at high cost, limiting the ability of other researchers to replicate the approach elsewhere or for research to continue after grant funding was expended [17].

As the spatial resolution of civilian satellite imagery steadily improved from 2-meters in the 1990s to 31-cm or better today, alongside ever expanding access to free imagery through web-mapping services such as Google Earth and Bing Maps, other researchers began to take advantage of these data to document looting and site damage (e.g., [18–20]). However, Google Earth and similar services do not update imagery very frequently, and therefore are of limited use in contemporary conflict zone cultural heritage monitoring [8].

Today, a growing number of archaeologists and heritage professionals have been able to gain access to high volumes of current, high-resolution satellite imagery, typically through partnerships with government agencies or private foundations. The majority of researchers use satellite imagery to perform site-based studies, designed primarily to either verify damage reported elsewhere [5,13,14] or to raise awareness of cultural heritage issues by highlighting damage to well-known sites (e.g., [7,9,10]). However, as barriers to imagery access are reduced, some research groups have begun to use imagery to conduct more systematic, regional-scale efforts akin to Stone’s [16], but covering larger regions, with improved spatial and temporal resolution, and more seamless integration into GIS platforms. These projects include the Endangered Archaeology of the Middle East and North Africa (EAMENA) organized through a consortium of British universities [21], the TerraWatchers initiative based at the University of California San Diego [22], the GlobalXplorer program, funded by the TED organization and hosted at the University of Alabama [23], the Afghan Heritage Mapping Partnership (AHMP) at the University of Chicago’s Oriental Institute [24], as well as our colleagues at the ASOR Cultural Heritage Initiatives (CHI) who have expanded efforts into North Africa and elsewhere [5]. With the proliferation of these and related projects, a new set of challenges have emerged, including questions surrounding the construction of site databases, efficient and effective documentation of damage, querying and making sense of results, as well as sharing and distribution of findings.

## Methods

The research project we report herein, undertaken as a component of the larger ASOR Cultural Heritage Initiatives, emerged out of an initial urgent need to evaluate the extent and severity of damage to archaeological sites in the context of the Syrian war, and therefore many of the decisions we made in the design and implementation of the project was driven by this exigency. The work builds on a pilot study undertaken in 2013–2014 that used only free and donated imagery resources [8], in which 40 key sites in Syria were evaluated for looting and other forms of damage. After receiving funding in 2014–2015 through a cooperative agreement with the US Department of State and ASOR, we planned to purchase imagery for 400 additional sites, but soon after the project began, our team gained access to a vast archive of continually updated DigitalGlobe imagery dating back to 2007, offering an unparalleled opportunity to institute a larger-scale monitoring effort. Given that only one year of support was guaranteed, we quickly sought to develop a strategy to survey systematically a sample of the thousands of sites in the region and to record observations in a manner that would facilitate spatial and temporal queries designed to answer key questions. Specifically we hoped to determine how many sites were being damaged and how severely, when this damage occurred, and where in Syria these sites were located. In so doing we could answer questions such as: Are sites of particular periods or types more commonly targeted by looters? Is looting correlated with other forms of damage resulting from militarization, construction, or agriculture? Is site damage more common in some parts of Syria than others, correlated with areas under control of ISIS or other political factions, with the intensity of military conflict, or with proximity to population centers? How has the severity of damage changed since the war began or evolved during its course?

Because our project was focused specifically on assessing war-related site damage, we focus on the period from 2010–2017, and in the first year of the project (2014–2015) only on Syria. We received a second year of support in 2015–2016 during which time we expanded our efforts into neighboring areas of northern Iraq, southern Turkey, and northern Lebanon and developed more systematic sampling strategies. Below we review our approach to developing a regional-scale monitoring system, including construction of an archaeological site database, its integration with DigitalGlobe satellite imagery resources, and our protocols for assessments of damage, logging of observations, and querying results. Note that because research presented herein relies exclusively on analysis of previously published archaeological site data and publicly available satellite imagery, no permits or special permissions are required.

### Archaeological site database

**Fig 1:**
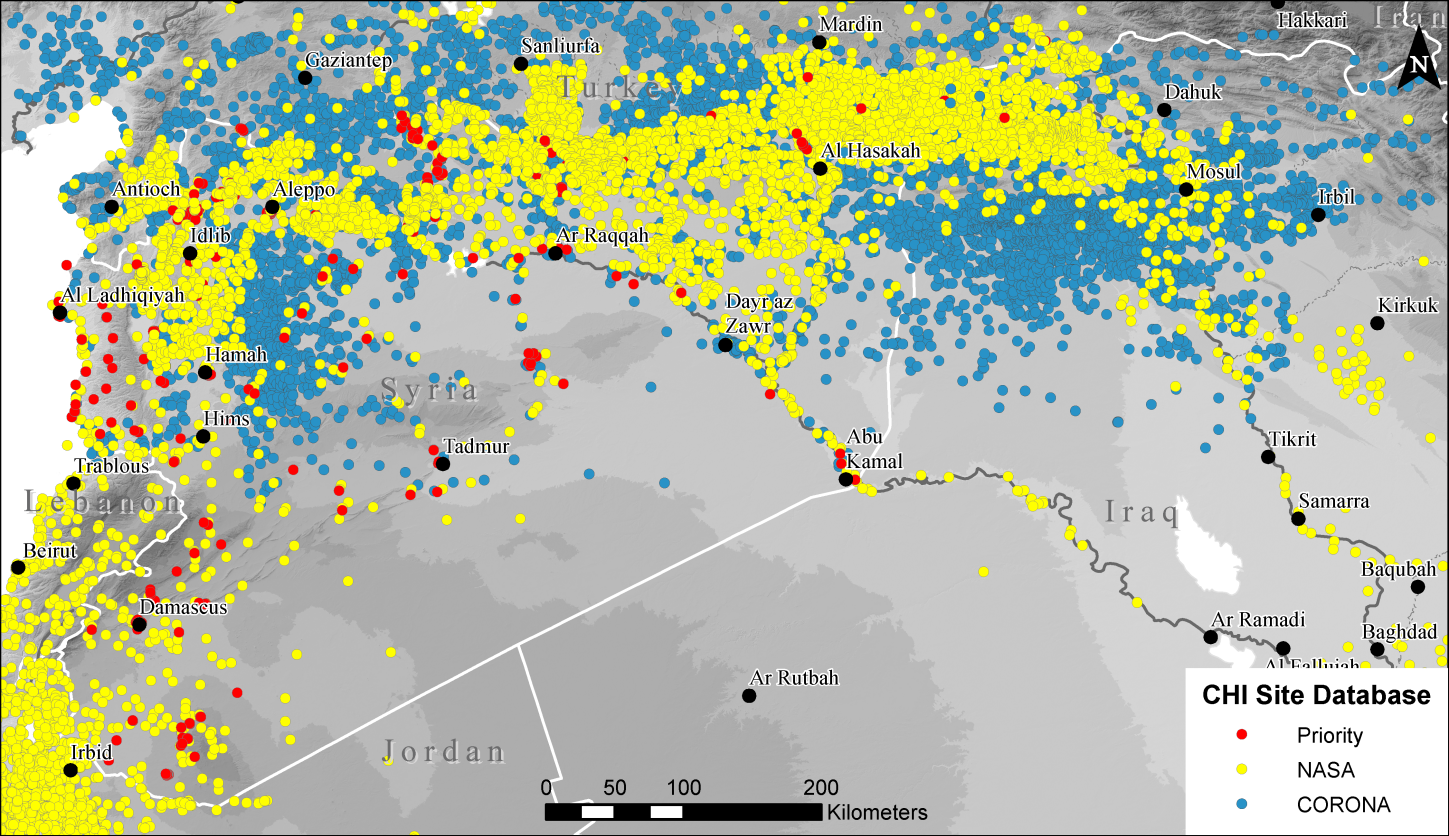
Map of known archaeological sites within the study area. Sites are classified as Priority “PRY” sites which have been excavated or are otherwise well-known, NASA sites which have been mapped from published archaeological surveys, and CORONA or “CRN” sites, which have only been documented using satellite imagery. Background SRTM DEM courtesy of the U.S. Geological Survey.

One of the most important elements in developing a comprehensive, regional-scale, imagery-based monitoring program is an archaeological and heritage site database (Fig 1). Our work benefits from a large site database we had developed previously through a NASA-funded research project [25–27]. Our existing database was designed to map primarily loci of ancient settlement, including mounded sites, architectural ruins, and dense artifact scatters. It also includes other features such as ancient cemeteries, ritual installations, or monuments, all of which are characteristically classified as archaeological sites in academic literature and by local governmental antiquities authorities. Focusing on northern Syria, southern Turkey and northern Iraq, we began building the site database by integrating data from several major atlas and mapping projects, including all sites in the Oxford Encyclopedia of the Ancient Near East [28], the PLEIADES Atlas Project [29], and the Digital Atlas of the Holy Land [30]. Because these kinds of large atlas projects typically report primarily either the best known sites, sites of a particular period, or sites in a given region, we next assembled forty previously published archaeological survey projects. Archaeological surveys are field projects that seek to document systematically archaeological sites and features within a study area, typically covering 25–500 square kilometers, although how sites are identified, the intensity of investigation, and detail and quality of published maps and other data varies a great deal across projects. Nonetheless, for each survey we first georeferenced published maps and then plotted the location of each site by reference to satellite imagery. Sites or features for which the location cannot be verified on satellite imagery or by other methods are excluded from our analysis, with the idea that if we cannot see or known the exact location of an archaeological feature, any further satellite imagery-based analysis is impossible. Collectively, sites known from both atlases and surveys produced a sample of around 4200 sites [25–27].

Because large parts of our study area have either never been subjected to systematic archaeological survey, or the results of such surveys have yet to be published, we supplemented the existing dataset of known sites with probable archaeological sites documented on 1960s-era CORONA satellite imagery. CORONA has been shown to be a uniquely valuable resource for identification of archaeological sites in the Near East [31,32], and enables a large percentage of likely sites to be mapped with some degree of confidence. Our work has added close to 10,000 previously undocumented or unpublished sites to the dataset [25–27]. Because some features we identify as sites on CORONA imagery are indisputably ancient settlements but others are more ambiguous as to whether they are cultural or geologic features, we also include a confidence indicator of “definite,” “probable,” or “possible,” that is made by the analyst who initially identified the feature and double checked by the project director or senior staff. Because archaeological field documentation of sites in Syria and other parts of the Middle East is highly inconsistent, with some areas virtually unexplored, a dataset representing only published and recorded sites would exclude the majority of sites in Syria. Finally, we incorporated several hundred additional heritage sites, including museums, historic buildings, libraries, and other monuments from a list developed by the International Committee of the Blue Shield.

Our resultant site database would be imperfect as a cultural heritage management inventory, as it excludes sites and features that are not resolvable in satellite imagery, and also includes many sites and features that are undocumented by archaeologists or antiquities officials. However, it forms a strong basis for our remote sensing-based research because it provides a fairly comprehensive sample of all archaeological sites (and probable sites) visible in satellite imagery. In the first year of our project (2014–2015) there was a great deal of urgency in our efforts to document potential damage to the best-known sites in Syria, as previous analyses and media reports had highlighted potentially severe looting and war-related damage at numerous UNESCO World Heritage or other popular touristic sites such as Apamea, Krak des Chevialers, and the so-called “Dead Cities” [11]. Thus, we began by establishing a subset of sites in Syria which we designated as “PRY (“priority”), not intended to signify which sites are most important, but rather simply those that are best-known to archaeologists and heritage professionals, and thus a means to create an initial sample of our site database that would best reflect the rapid, information-gathering goals of the project. We included in our “Priority” list all sites reported in two key sources, *The Archaeology of Syria* [33] and *Monuments of Syria* [34], which collectively capture nearly all excavated sites dating from 18000–300 BC, as well as all later sites with standing architectural remains. We supplemented this list with other key historic buildings and monuments not reported in these books, as well as several other sites that have more recently been excavated or published.

**Fig 2:**
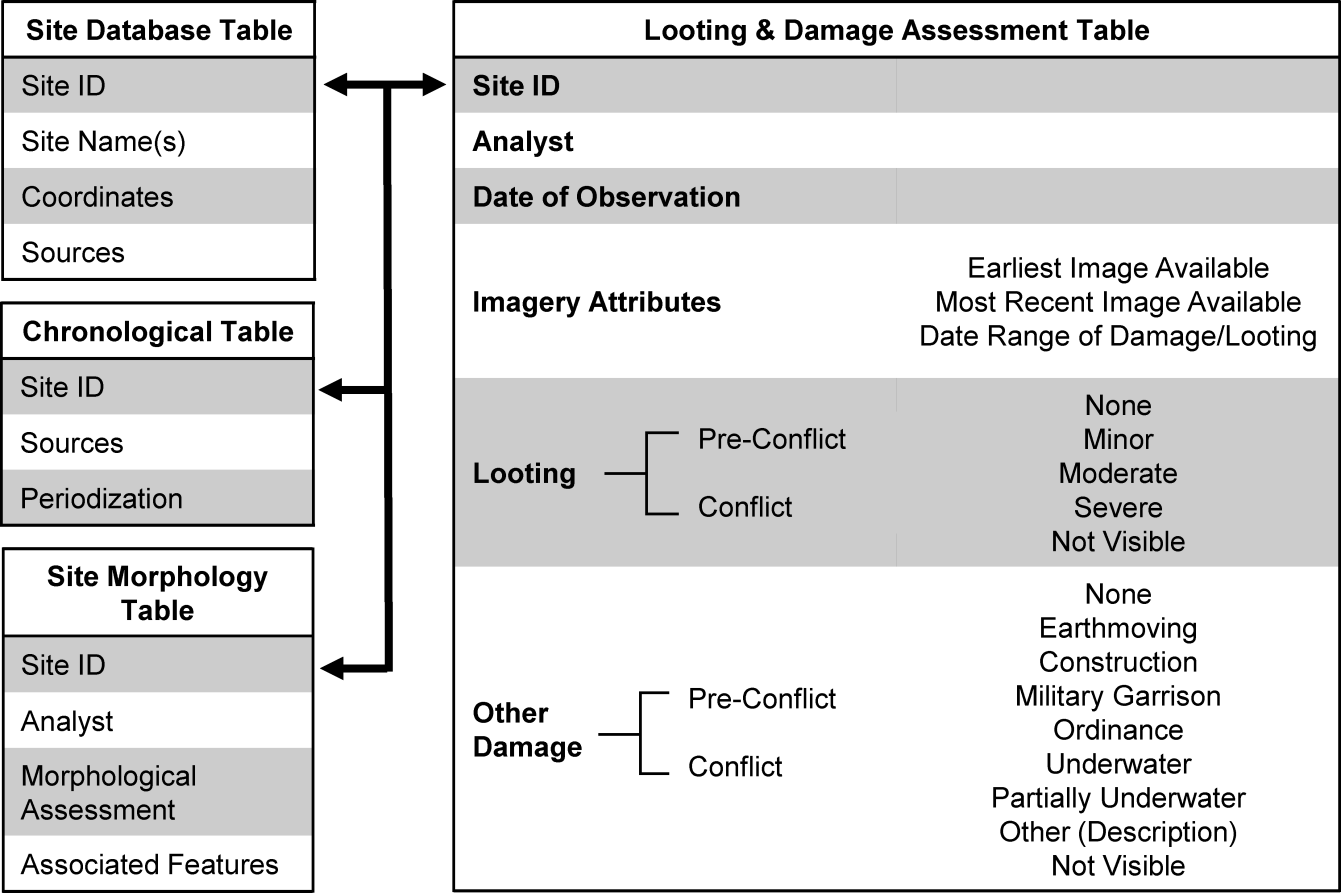
Structure of site and assessment database.

The site database includes unique identification numbers for all sites, each with a designator to quickly enable sorting in terms of priority and certainty, including Priority “PRY” sites, “NASA” sites, which are those for those mapped from archaeological survey reports, and “CRN” (CORONA) sites known only from imagery-based analysis (Fig 1). Our site database contains several distinct tables all linked via sites’ unique PRY, NASA, or CRN numbers (Fig 2), allowing queries to be structured across tables as necessary. Site locational data, names, descriptions, and bibliographic citations are all contained in a primary table, while a second table records periodization as reported for published sites, and a third table records site morphology as visible on CORONA imagery [25,26,35]. For the purposes of this project, we created a fourth table to record observed damage to sites, and later added a fifth table to record the complex issue of the timing of damage, both described in detail below. Collectively, a database structured in this manner allows us to design spatio-temporal queries across domains in order to answer key questions (e.g, plot all of the mounded, Early Bronze Age sites located in ISIS territory that have been looted since 2014).

### Damage assessment protocols

Our initial pilot study undertaken prior to the start of the ASOR CHI project [8] enabled us to have a clear understanding of the types of damage we were likely to encounter in imagery-based assessments, including not only looting, but also militarization, earthmoving, construction and other issues. In order to scale-up our methods, we streamed DigitalGlobe imagery directly into the ArcGIS interface using DigitalGlobe’s ArcGIS plug-in, ImageConnect. Analysts simply open our site database in ArcMap, connect to the DigitalGlobe image server, and then are able to turn on and off individual satellite images as they log observations. For each site, we compare imagery from before the war (March 2011 or earlier) with the most recent image available. Any observed changes that can be seen are then logged into a damage assessment table. To facilitate use by multiple analysts at different institutions, individual users first access a central database housed on project server, create a local copy, proceed to log observations or make other edits, and then sync results back to the primary database.

### Identification of looting

**Fig 3:**
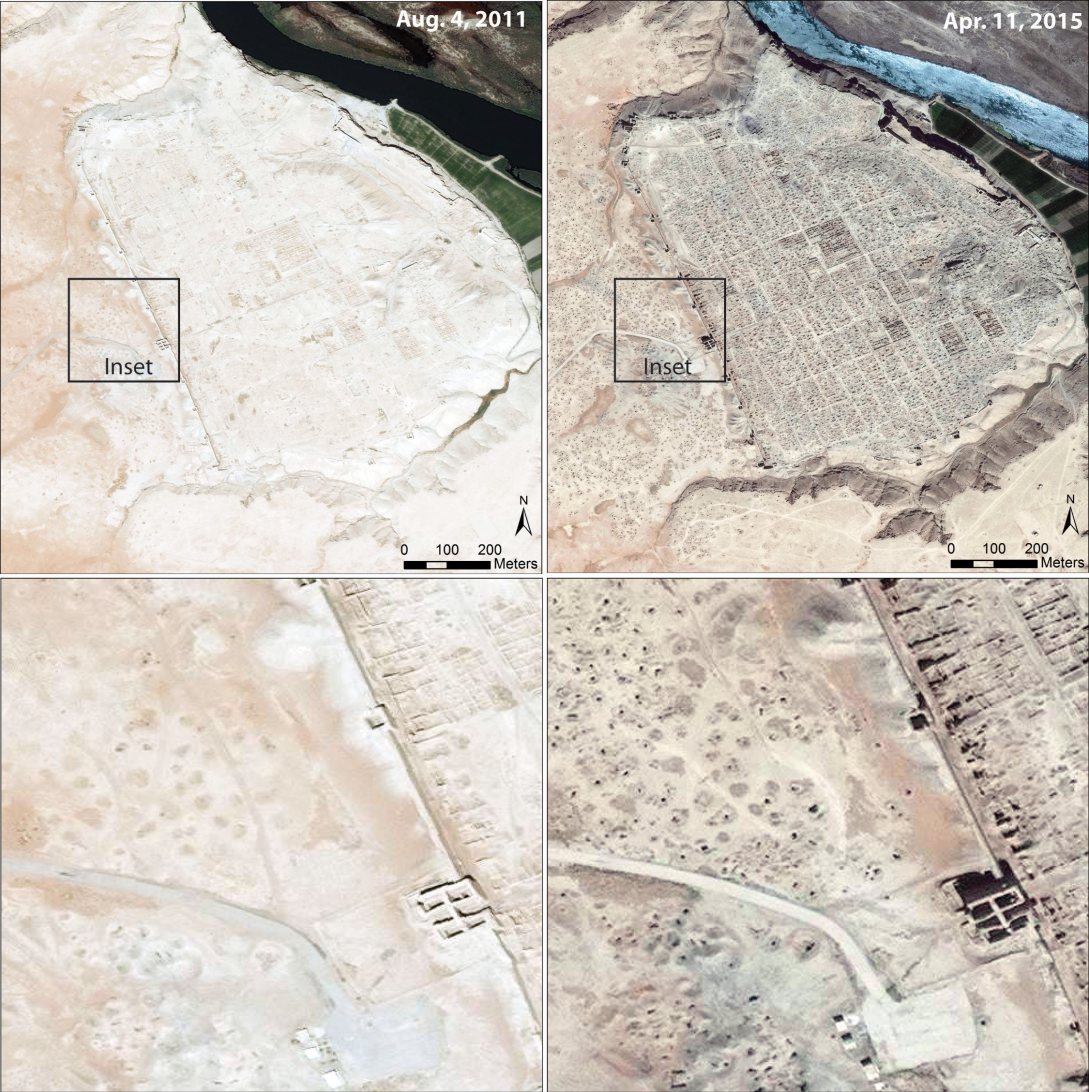
Looting at the Hellenistic/Roman site of Dura Europos, eastern Syria. Imagery from August 2011 (left) reveals a decades-long history of looting outside the Palmyrene Gate. Imagery from 2015 (right) shows a renewed phase of severe, war-related looting. Satellite imagery printed under a CC BY license, with permission from DigitalGlobe 2017.

In our assessments, looting is most commonly recognized by the presence of holes dug on archaeological sites, which can form fairly conspicuous features in satellite imagery—each hole appearing as a dark spot, usually 1-3m in size, with an adjacent upcast mound. The vast majority (>99%) of looting holes are sub-rounded or amorphous in shape, making them relatively easy to distinguish from construction trenches, military bunkers, or other forms of excavation on sites. Older looting holes typically erode into sloped craters, while completely eroded holes may appear as a dark spot on the ground. In some cases, looting holes can remain visible for decades, as is the case at Dura-Europos, where a long history of looting is visible outside the Palmyrene Gate in imagery from 2011 (Fig 3). Imagery from April 2015 shows a renewed phase of intensive looting during the war, with hundreds of new holes visible, sometimes even inside older looting trenches. In other instances, as at Tell Cheura, rather severe looting that appeared in 2013 [8] had been almost completed obscured in the following year. Thus, when we classify looting at a site as “none” we simply mean we are unable to see any looting; many of these sites may have been looted in the past, but this cannot be determined solely on the basis of satellite imagery.

In some cases, ground conditions such as dense vegetation, modern construction, or flooding below reservoirs may make it impossible to determine whether looting has occurred. Similarly, inadequate imagery coverage, or cloud-covered imagery, may make assessments impossible. In these cases we classify sites as “Not visible” such that when structuring a query to evaluate the percentage of sites that have been looted, we can exclude sites where we are unable to make a determination, keeping in mind that a positive assessment of looting still carries more analytic weight than a negative determination. Finally, we have consistently found that inexperienced observers often confuse orchards, haystacks, cemeteries, or other features for looting holes, and so our assessments are all conducted by trained analysts and double-checked by senior team members (the authors of this paper).

### Severity of looting

Determining the severity of looting in satellite imagery remains a complex problem, with some previous researchers seeking to measure the looted area of sites (e.g., [15,16]), others seeking to count individual looting holes (e.g., [36]), and still others creating complex classification schemes (e.g., [37]). Each of these methods has strengths and weaknesses. For example, measuring looted area requires a judgment about the density and spacing of looting trenches, while counting individual holes does not take into account their variable depth and size; moreover, all of these methods are highly time intensive. Recent attempts to automate detection of looting holes using spectral analysis of high-resolution full-spectrum optical imagery [38,39] or textural analysis of topographic data derived from space-borne radar [40,41] are promising, but not yet applicable across large regions due to the high cost of appropriate imagery.

In an effort to balance a rapid observation strategy with the need to record the scale of looting at individual sites, we elected for a simplified classification scheme of minor > moderate > severe to describe looting severity. As a general rule, sites with fewer than 10–15 looting holes, depending on the size of the site and the spacing of the holes across it, are considered “minor,” while those with either a large number or a significant percentage of the site having been looted are considered “severe.” As with any classification scheme, there are many cases in which individual analysts will disagree as to whether they should be classified as “moderate” or one of these other two categories. However, the creation of a disputed middle category means that we have no disagreement among sites that are “minor” versus those that are “severe.” Moreover, the system enables assessments to be done quickly, and the total number of looted sites are not so large than they cannot be individually reviewed.

### Assessing the timing of damage

**Fig 4:**
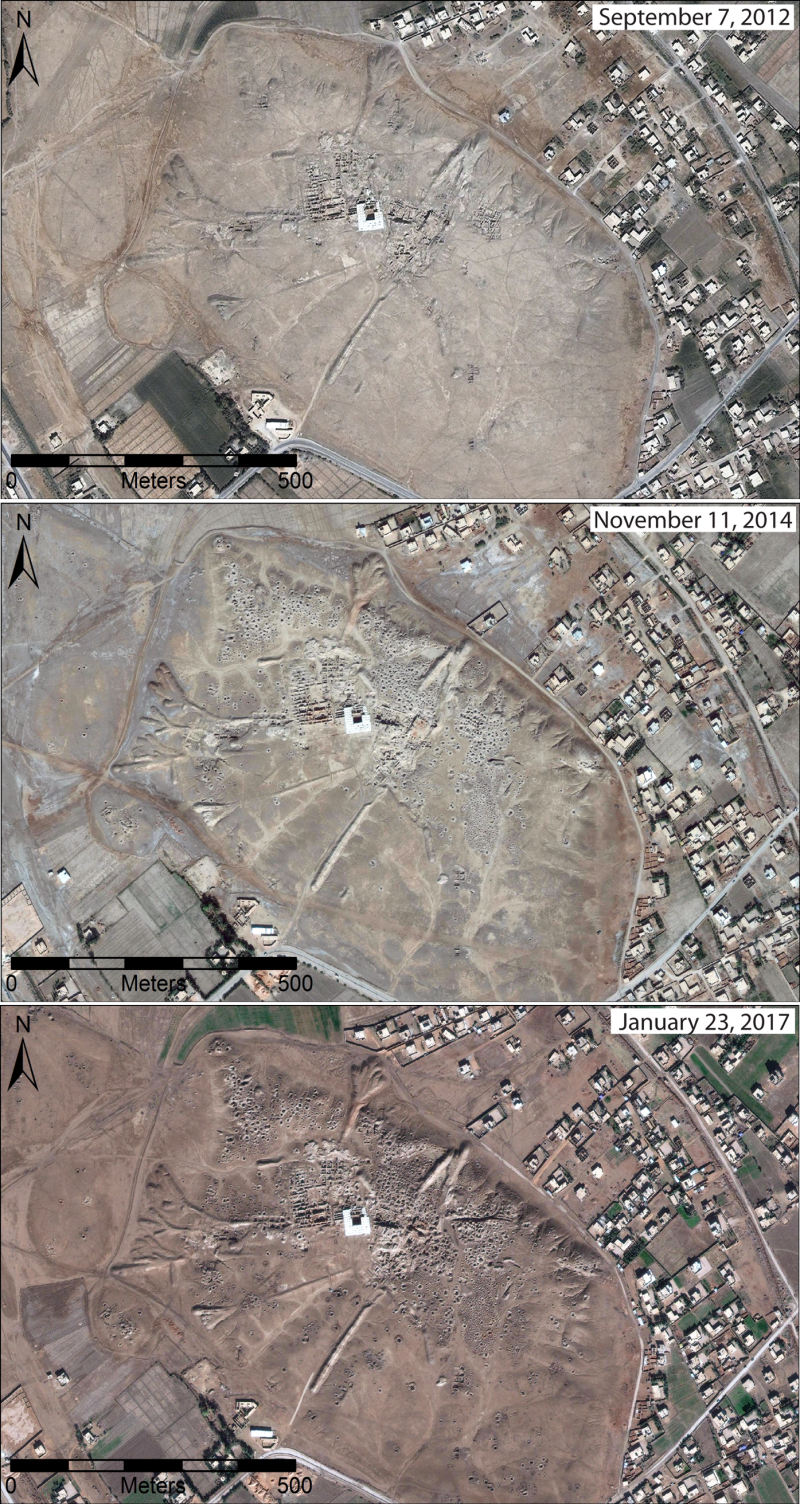
A time series of images of Mari, a Bronze Age city in eastern Syria. The sequence shows that severe looting devastated the upper mound sometime between September 2012 and November 2014, with moderate looting continuing through January 2017. Satellite imagery printed under a CC BY license, with permission from DigitalGlobe 2017.

Determining when looting has taken place is also a complex problem, mostly due to the highly variable imagery coverage of individual sites (Fig 4). Some sites in our database have dozens of different images, spaced evenly over several years, in which case we could carefully track when particular episodes of looting or other kinds of damage took place. However, because imagery collection is driven primarily by political and military events on the ground, there are occasionally sites for which we have dozens of images available over a relatively short period of time, bracketed by periods of up to several years during which no imagery is available. Most sites have only a few available images, and many may have only two separated by up to five years. This means that in some cases we can determine the specific month in which an episode of looting or damage took place, while in other cases we can only say that it took place sometime within a 5-year window.

In our initial construction of our database, we considered temporality in a binary way, recording only whether it took place before or since the war began, enabling us to quickly identify war-related damage. However, we have also developed a method for assessing the probable frequency and rate of looting events in a more fine-grained manner, which accounts for the inherent temporal uncertainty driven by imagery availability. First, for all sites at which we observed war-related looting, we gathered all available DigitalGlobe-served images. Next, we carefully documented when looting episodes were visible in each image, enabling us to determine the time-range in which each looting incident could have occurred. We then assigned numerical values to the severity of cumulative looting at each site (i.e., severe = 3, moderate = 2, and minor = 1). Next, we simply divide the cumulative index of looting severity for a site (1–3) by the timeframe in months over which the looting incident could have occurred, as documented by temporally bracketed satellite images. This calculation results in an average probability that an episode of looting occurred in any individual month across the entire timeframe. For example, if the best we can determine is that a site experienced minor looting, with a numerical value of 1, at some point within a 10-month period, then we assign a 10% chance that the looting took place within in any of those 10 months, and thus each month is scored as 0.1. In contrast, for a site that experienced severe looting across a 2-month period, each month is given a 50% probability, and a score of 1.5. Each site was given a cumulative looting severity score instead of each individual looting episode so as not to give greater weight to sites with more imagery availability (problematically greater imagery availability can lead to more possible discernable episodes of looting). Finally, we then simply sum the total probable looting scores for each month to derive a relative index of looting frequency. This approach is similar to that proposed by Dewar [42] for calculating the likelihood that sites were contemporaneously occupied when dealing with *terminous post* and *ante quem* data for archaeological periods of differing length. Our method is imperfect in many respects; for example, weighting cases of “severe” looting as three times the value of “minor” looting is a rough estimate of their relative severity. However, changing these values, for instance designating “severe” as ten times the value of “minor, would simply exaggerate the same trend line that our process produces, and so we believe our approach has value in offering a perspective on looting incidents and severity over time.

### Assessing other forms of damage

For the most part, assessments of other kinds of damage are more straightforward than assessments of looting. New construction, earthmoving, or the expansion of cemeteries on sites are all relatively easy to recognize in imagery. In some instances it may be difficult to distinguish between earthmoving undertaken for construction or agricultural purposes versus that undertaken as part of a site’s militarization, but typically military bunkers appear as distinct linear trenches, and are often associated with tents, vehicles, and other activities on sites. Occasionally we have encountered forms of damage that are difficult to classify within our scheme, and these are labeled as “Other” in the damage fields. As with looting observations, we also include a not-visible category for instances when imagery availability or quality, or ground conditions, prevent a damage assessment.

## Results

At the conclusion of our funded project in September 2016, our team had undertaken assessments at 4922 sites, including 241 Priority sites that have been excavated or are otherwise well-known (PRY), 2860 sites known from survey publications and gazetteers (NASA), and 1821 sites known only from our own satellite imagery-based mapping (CRN) (Table 1). Assessments include 3391 sites in Syria, 851 sites in the Ninewa and Anbar provinces of northern Iraq, 633 sites in southern Turkey, and 47 sites in northern Lebanon. While the first year of the project was dedicated exclusively to Syria, in the second year we expanded our analysis to northern Iraq, and finding significant differences across the national border, also decided to build a comparative sample from Turkey and Lebanon for baseline comparisons.

**Table 1:** Site assessments by country and database type.

	Total Assessments	Database Type		
Country		NASA	PRY	CRN
**All Countries**	4922	2860	241	1821
**Syria**	3391	2274	241	876
**Iraq**	851	240	n/a	611
**Turkey**	633	299	n/a	334
**Lebanon**	47	47	n/a	0

DigitalGlobe did not provide imagery of sufficiently recent date to make a wartime damage assessment at the time we evaluated all sites in our database, and thus queries must take into account these availability and visibility factors. Over the two years of the project, imagery for many sites has continued to be updated, and we have made efforts to balance the need to update older site assessments alongside expanding new ones. In addition, our sampling strategy evolved over the course of the project. In the first year, we focused on completing assessments at all priority sites and other well-known sites before moving on to a random sampling of other sites, largely driven by the pressing desire to know the status of key sites. In the second year of the project, we specifically focused new assessments in areas with relatively recent imagery in order to ensure we were capturing the most current situation on the ground. These differences in sampling are reflected in our results, as data from the second year includes a larger percentage of smaller, less-well known or completely undocumented sites.

Of the 4602 sites where imagery postdating 2011 was available at the time our assessment was undertaken, 1808 had imagery available dating to 2016, 1023 sites were imaged in 2015, 1285 in 2014, 410 in 2013, and 76 in 2012. Thus, 89% of our observations are based on imagery from 2014 and later, and more than 50% are from late 2015 to the present. Below we outline key findings in terms of looting and other forms of damage.

### Looting

**Fig 5:**
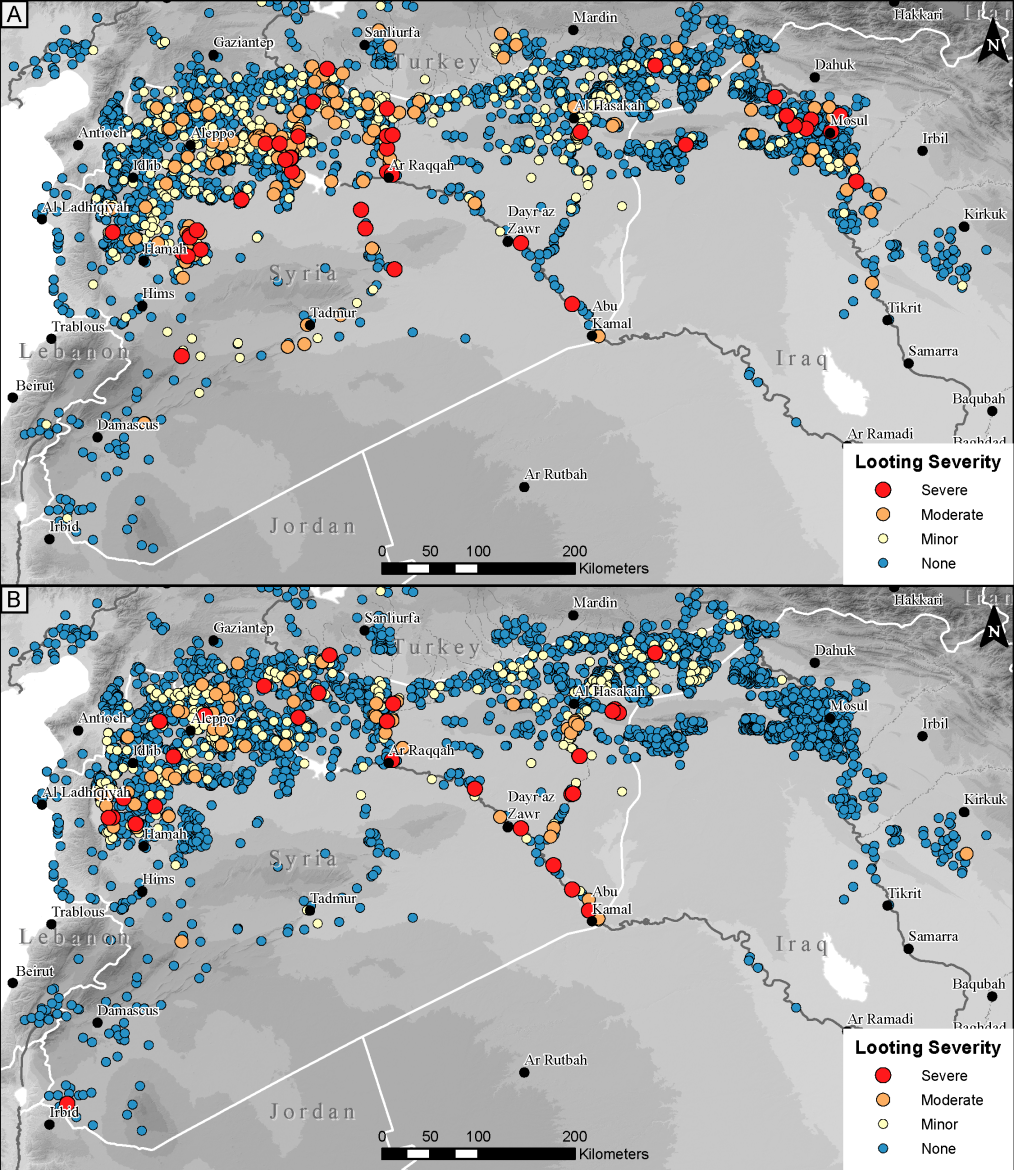
**Documented pre-war looting (A) and war-related looting (B).** Severity of documented looting predating (A) and post-dating (B) the beginning of the current Syrian war in March 2011. Background SRTM DEM courtesy of the U.S. Geological Survey.

Our analysis shows that of the 3909 sites in our database where it has been possible to make looting assessments both before and since the war in Syria began (i.e., sites where imagery of sufficiently old and sufficiently recent date is available, and where sites are not obscured by modern land use or land cover), 13.5% (n = 529) show evidence of pre-war looting, whereas 10.7% (n = 363 sites) have been looted since the war began in mid-2011 (Fig 5; Table 2). In terms of the severity of war-related looting, 28 sites were classified as having severe looting, 54 sites with moderate looting, and 281 sites with minor looting.

**Table 2:** Summary of site looting by severity, database type, and country.

	Total Assessments	Database Type			Pre-War Looting		War-Related Looting					
Country		NASA	PRY	CRN	Count	%	Count	%	Minor	Moderate	Severe	None
**All**	**3909**	**2142**	**203**	**1564**	**529**	**13.53**	**363**	**10.77**	**281**	**54**	**28**	**3452**
**Syria**	3641	1703	203	735	450	17.04	355	13.44	276	52	27	2197
**Iraq**	825	233	n/a	592	50	6.06	2	0.24	1	1	0	823
**Turkey**	424	187	n/a	237	28	6.60	6	1.42	2	1	1	413
**Lebanon**	19	19	n/a	0	1	5.26	0	0.00	0	0	0	19

All columns are site counts except where specified as percentages (%). % is percent of total pre-war or war looting for each country.

These numbers suggest somewhat lower overall rates of looting than our previous analysis undertaken in 2014–2015 [6], but is largely due to the major differences we observe across national borders. Broken down by country, Syria shows by far the highest rates of looting, with 17.0% of sites (n = 450) with evidence of looting prior to the war, and 13.4% of sites (n = 355) looted since the war began. In comparison, in our sample of 825 sites in northern Iraq, only 50 (6.1%) show evidence of pre-war looting, and a strikingly low 2 (0.2%) appear to have been looted within the past five years. A similarly low percentage of both pre-war and post-2011 looting is seen in the sample of 424 assessable sites in Turkey, where 28 sites (6.6%) show evidence of pre-war looting and only 6 (1.4%) have evidence of post-2011 looting.

There is no immediately evident explanation for the extreme differences in rates of both pre-war and post-war looting across national borders. There are not major differences, for example, in population density or distribution, the type or period of sites represented in the sample, or the modern land use and land cover conditions. And notably, rates of looting in northern Iraq appear to be considerably lower than those documented in southern Iraq, where particularly during the mid-2000s following the 2003 US-led invasion, looting reached extreme levels akin to those seen in recent years in Syria [15,17].

Since at least 2014, media reports and public statements by government officials have largely focused on the role that ISIS has played in the larger cultural heritage crisis; certainly the terrorist organization has been uniquely diabolical in some respects, including publicizing intentional demolition of ancient and religious monuments [4,5] and, despite some skepticism over the monetary value of artifacts, purportedly sanctioning the looting of archaeological sites for profit [43,44]. ISIS became so closely associated with looting and illicit sale of antiquities that even in cases where objects are known to derive from outside ISIS territory, such as a mosaic from Apamea featured in a CBS investigation that was likely looted under the watch of the Syrian military, reporting draws a connection to ISIS [45]. Similarly, reporting on Hobby Lobby’s illegal purchase of cuneiform tablets, which originated in southern Iraq and were likely looted years before the war in Syria began, often links the sale to ISIS [46,47]. Our previous work [6] sought to counter these media narratives by highlighting the widespread nature of looting and other forms of site damage across all parts of Syria, with most frequent looting incidents in the most anarchic parts of the country, and severe looting occurring on sites occupied by the Syrian military.

**Fig 6:**
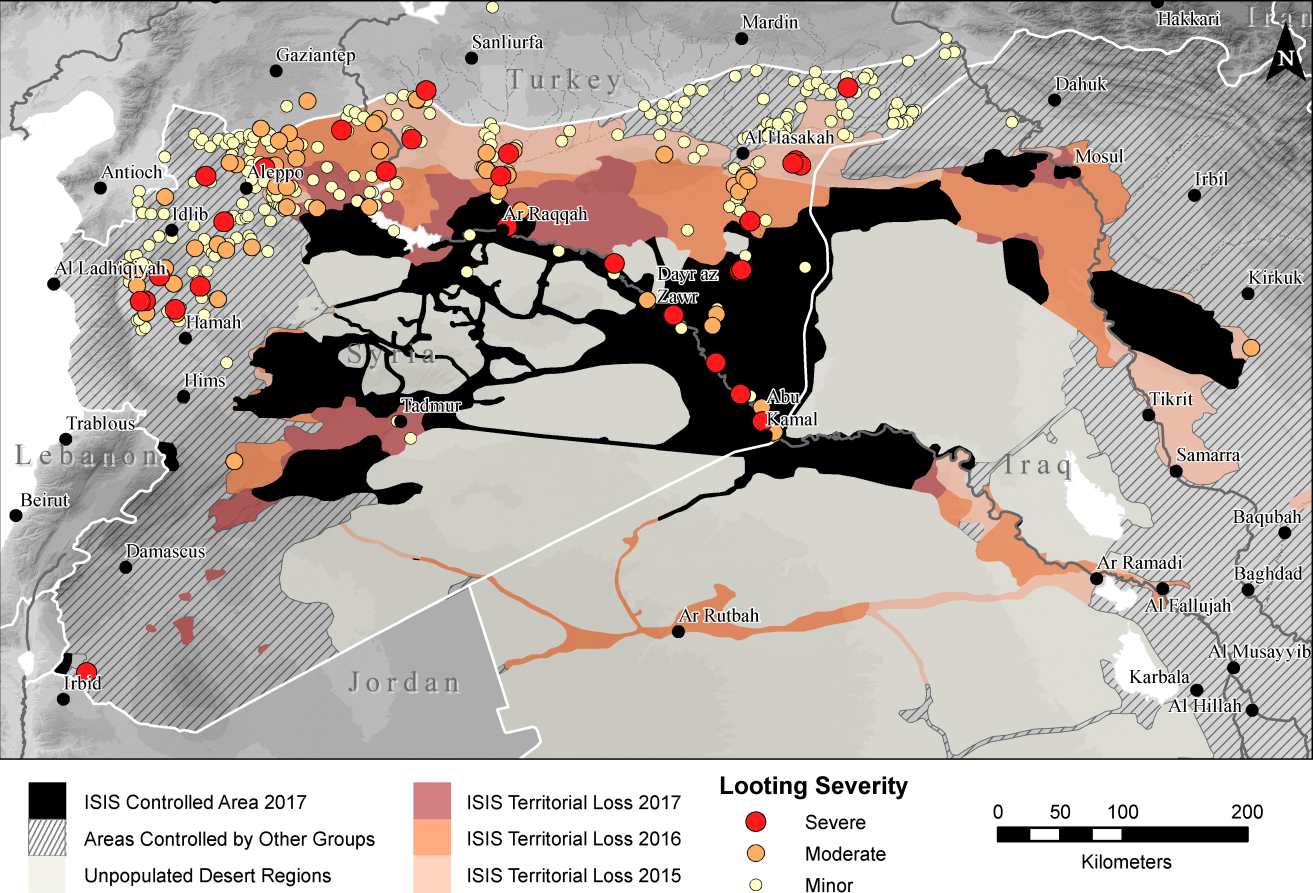
Map of documented war-related looting in relation to ISIS territory. Sites looted between 2011 and end of 2016 plotted against maximum extent of ISIS territory. Control areas modified from IHS Conflict Monitor (April 3, 2017) [48]. Background SRTM DEM courtesy of the U.S. Geological Survey.

Our new data continue to reveal a complex picture of the geographic distribution of looting when it is analyzed against areas under ISIS control (Fig 6). Determining specifically which areas are under which factions’ control has become increasingly difficult as the war has progressed, as areas of influence have shifted rapidly over the past several years. In this analysis, we illustrate all areas of Syria and Iraq that were ever under ISIS control, with progressive territorial losses from 2015–2017 as reported by IHS Conflict Monitor [48]. Our data show that looting is ubiquitous in the populated areas of war-torn Syria, and 50% of all looted sites are in areas that were never under ISIS control. On the other hand, many of the most severely looted sites are in areas controlled by ISIS along the lower Syrian Euphrates and in the Balikh Valley north of Raqqa. However, we see a similar concentration of severe looting in northwestern Syria, at sites controlled mostly by the Syrian military, as well as a few in areas held by opposition forces. These results continue to reiterate that war, more than any individual faction, is responsible for looting across Syria [6,49]. Furthermore, the near total absence of war-related looting in northern Iraq, even in areas that have been under ISIS control since 2014 and where there is a dense concentration of archaeological site quite similar to those found in Syria, illustrates again the degree to which localized factors drive looting.

Finally, our previous analysis of looting rates in Syria suggested somewhat higher overall numbers, with just over 20% of sites showing evidence of looting [6], as compared to 17.0% with evidence of pre-war looting and 13.4% with evidence of war-related looting in our new, larger dataset. These somewhat lower rates are driven primarily by the fact that our expanded dataset of 2641 sites in Syria includes a much higher percentage of “CRN” sites, which are sites that have not been published in surveys or gazetteers and thus are for the most part unknown to archaeologists or heritage officials. Because in the first year of the project we intentionally focused our efforts on documenting damage at better known sites, our 2015 looting assessments included only 38 CRN sites as compared to our current dataset which includes 735 CRN sites in Syria alone, and 1564 overall. These sites are often smaller, and topographically indistinct, which is part of the reason they have continued to go undocumented in the field. They are difficult for archaeologists to find, and seem to be similarly difficult for many looters to locate. Alternatively, smaller, flat sites may be perceived by looters as less likely to produce salable antiquities as compared to larger, more prominent, or better-known sites. Whatever the reason, analysis shows that 80% of looted sites form prominent mounds, while these sites as a category form less than 35% of sites overall, suggesting that mounded sites are considerably more likely to be looted.

### Timing of looting

**Fig 7:**
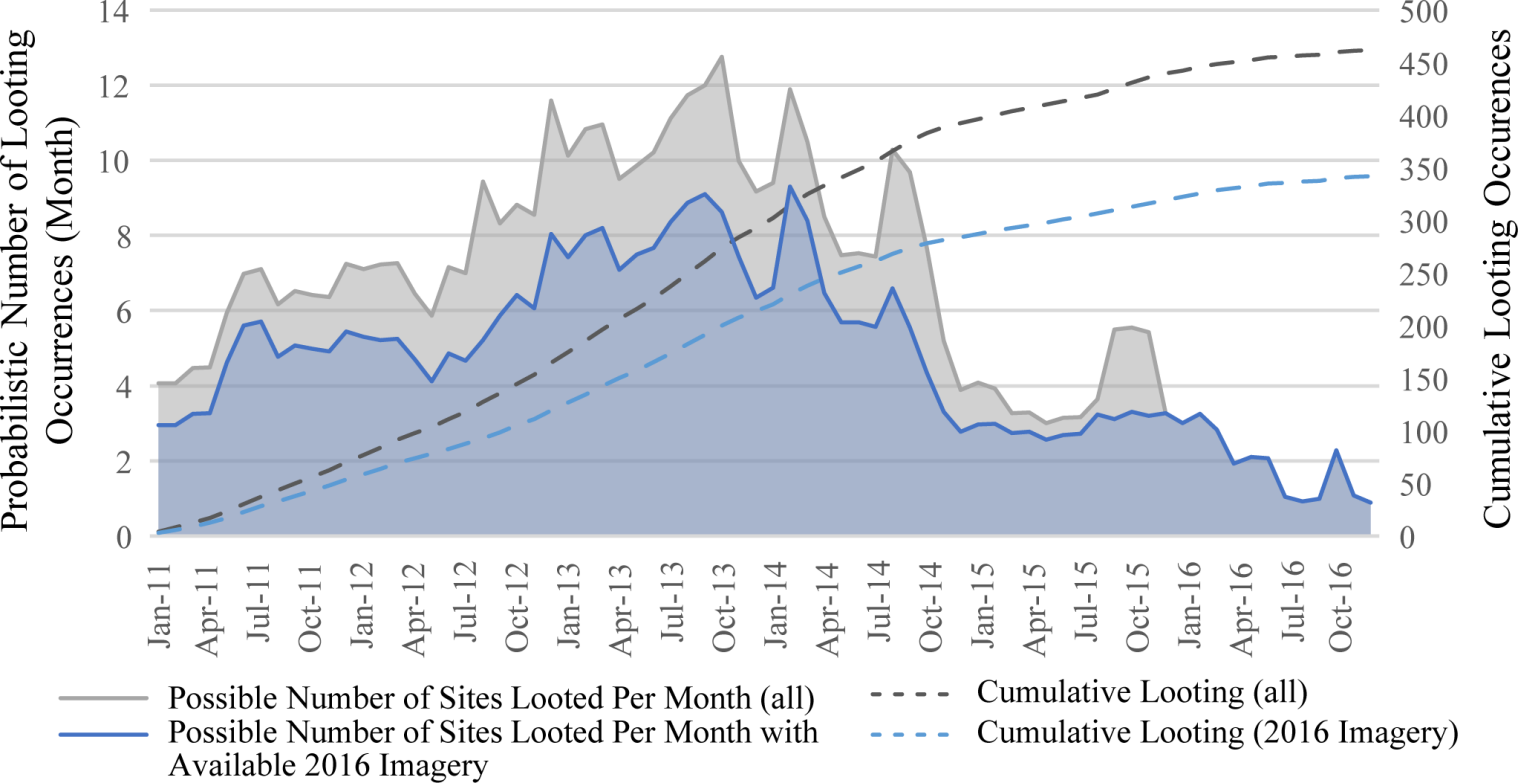
Trends in probability of looting by month. Chart illustrates the probability that looting incidents took place within any individual month, weighted by looting severity. Results show that looting rates began to decline in late 2014. Portions on graph in blue are a subset of sites where imagery was available through 2016.

As discussed above, we have developed a method to assess how frequently new incidents of looting are occurring by first determining when each individual episode of looting took place. Fig 7 illustrates results of this analysis for all sites where looting was observed through 2016. Note that in some cases where a large number of satellite images are available, looting incidents can be isolated to an individual month, while in other cases, we can only say that they took place sometime within a 5-year window.

We then calculate the probability that each individual looting incident took place within any given month (Fig 7). The results produce a trend line suggesting that since the war began in 2011, looting rates in Syria increased by an order of magnitude over pre-war levels, and continued at a steady rate until the last quarter of 2014 when they began to decline. From late-2014 to the end of 2016, overall incidences of looting decreased considerably, with the notable exception of the largest and most well-known sites where looting continued unabated. Because 89.3% of sites included in our looting analysis have imagery from 2015 or later and 70.7% of sites from 2016 or later, the trend line representing a decline in looting rates cannot be attributed to imagery availability or other sampling issues, but instead reflects a real decrease in both severity and frequency of looting incidents.

### Militarization of sites

**Fig 8:**
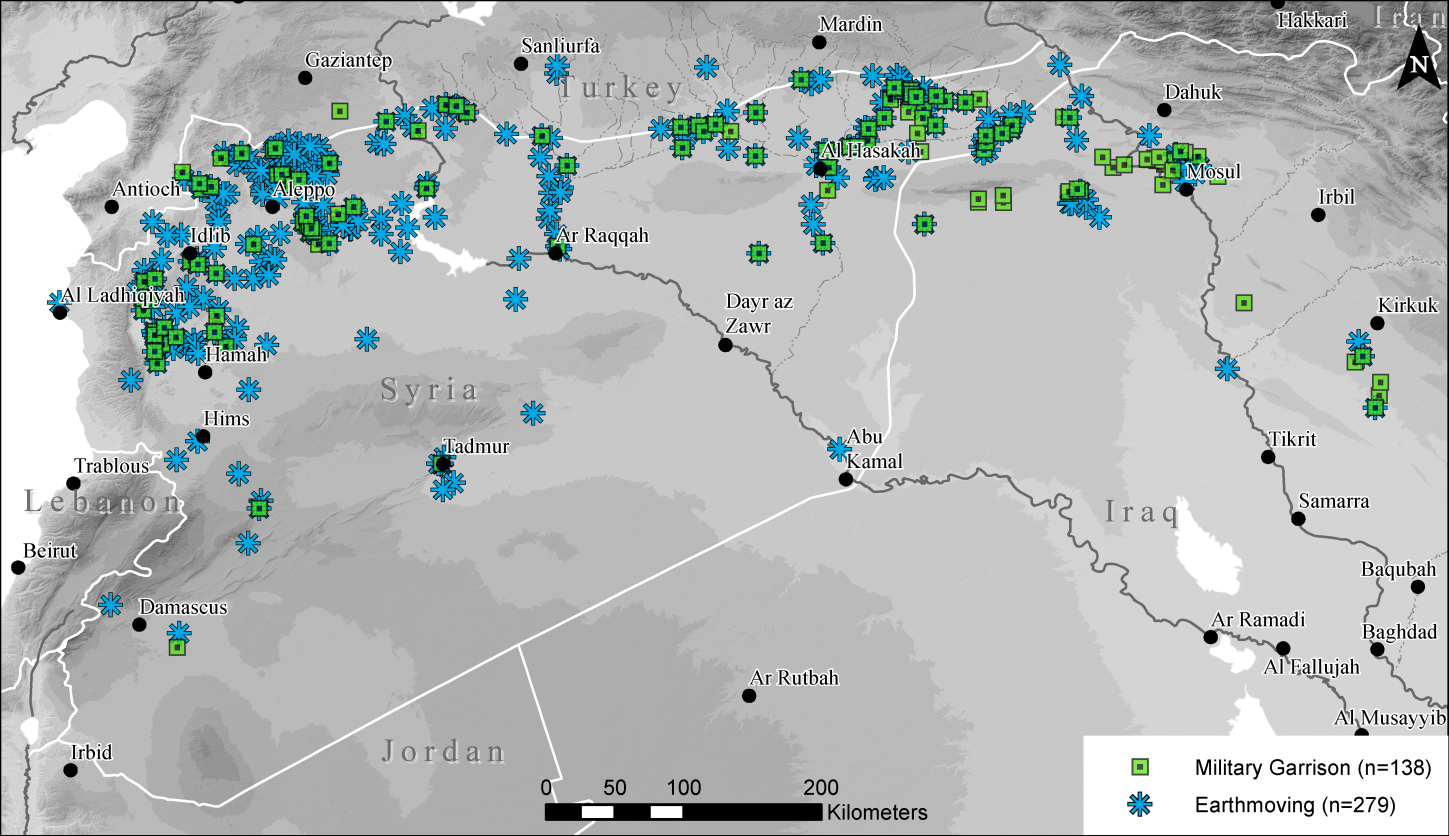
Map of observed militarization of archaeological sites. Map includes sites with full military garrisons as well as those with earthmoving that is likely related to military activity. Background SRTM DEM courtesy of the U.S. Geological Survey.

While the overall rate of looting in Syria may have waned from 2014–2016, and remains rare in northern Iraq, we have documented a steady increase in the number of sites that have been impacted by militarization on both sides of the Syrian-Iraqi border (Fig 8). Sites classified as having military garrisons typically include multiple lines of major trenching, installation of tanks, artillery or other heavy machinery, as well as temporary residence of soldiers or other personnel. A number of other sites are classified simply as having evidence of “earthmoving,” which is predominantly related to military occupation. All of these activities cause severe damage to archaeological sites, as it results in sites being strewn with trash, obscured by construction, and even made dangerous to future study by leaving behind unexploded ordinance or hazardous materials.

In our 2015 analysis, we documented only 2.5% of sites (n = 33) with evidence of militarization, all of which were in Syria. Our expanded dataset shows 3.5% of sites (n = 138) sites with military garrisons, and an additional 279 sites impacted by likely related earthmoving. While only a modest increase in percentage terms, we expected to see a *decrease* in our new sample because it includes a much higher percentage of smaller, topographically flat sites. Prominent mounded tell sites remain by far the most common type of site impacted by military activities, as they often form strategic high points in otherwise flat plains. Many of these tall mounds were indeed fortified during antiquity and are often situated in naturally strategic locations, making them particularly vulnerable to military-related damage during the current conflict. As discussed above, our 2016 data includes 37% CRN sites, which are sites known only from satellite imagery-based mapping, as opposed to our 2015 sample in which these sites constitute just 3%. CRN sites are most commonly smaller and less visible features in the landscape, and therefore less likely to be militarized. Thus, despite a dataset that includes a larger percentage of CRN sites, we see a rise in militarization.

The increase in militarization is most evident in northern Iraq. In Syria, our dataset of militarized sites for 2016 increased to 3.9% (n = 103), but in Iraq our sample of only 825 sites includes 53 (6.4%) will either full military garrisons or earthmoving that is likely related to militarization. Nearly all of this militarization has taken place over the past two years, and is concentrated in the conflict zone along the border regions between ISIL-held areas and the Kurdistan Region to the east.

**Fig 9:**
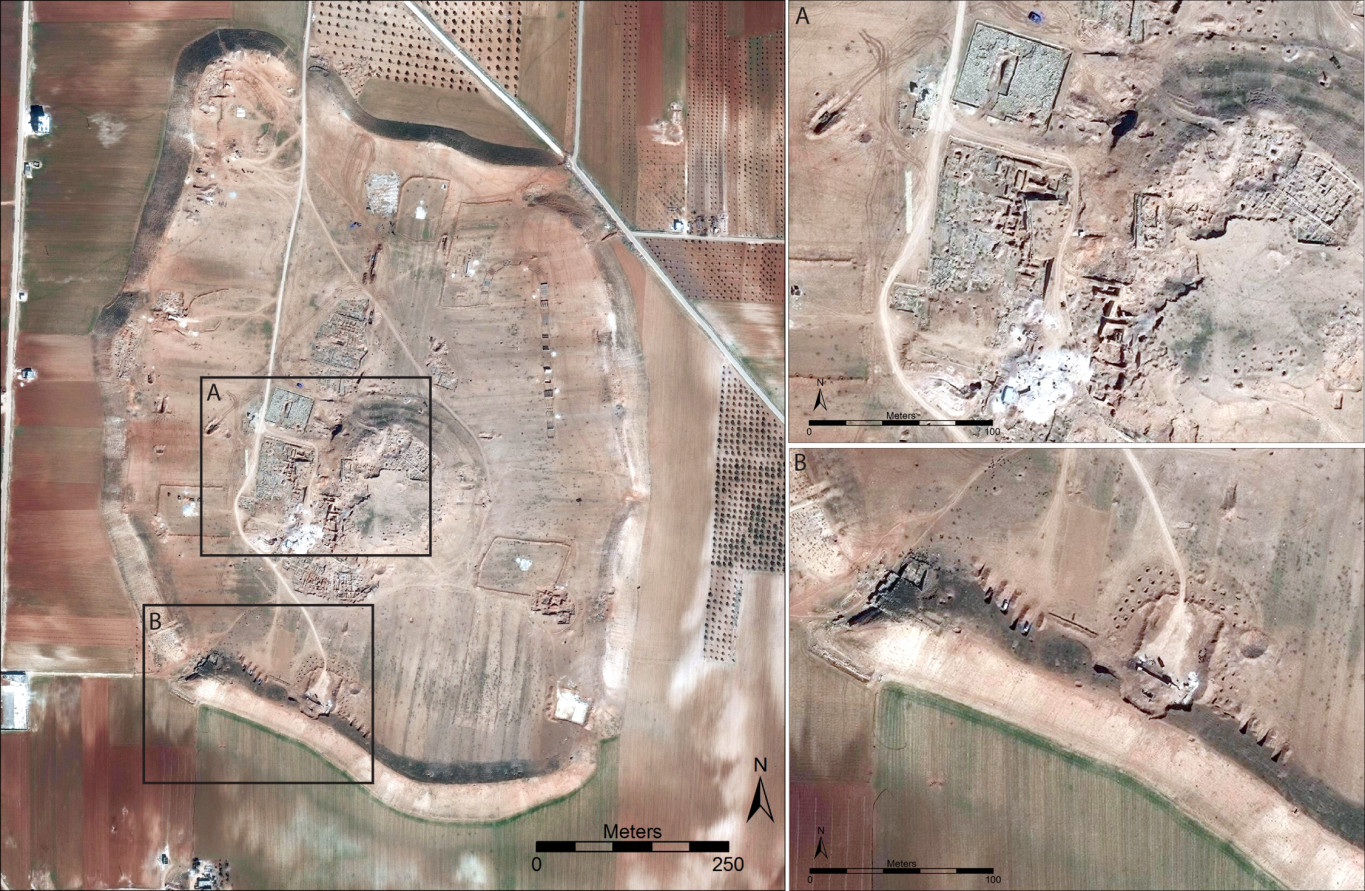
Military damage at the Bronze Age city of Ebla in western Syria. Militarization at Ebla began in 2013 [8] and has continued to intensify throughout the war, with severe looting around the central citadel (A) and earthmoving throughout the site (B) in this February 2017 image. Satellite imagery printed under a CC BY license, with permission from DigitalGlobe 2017.

**Fig 10:**
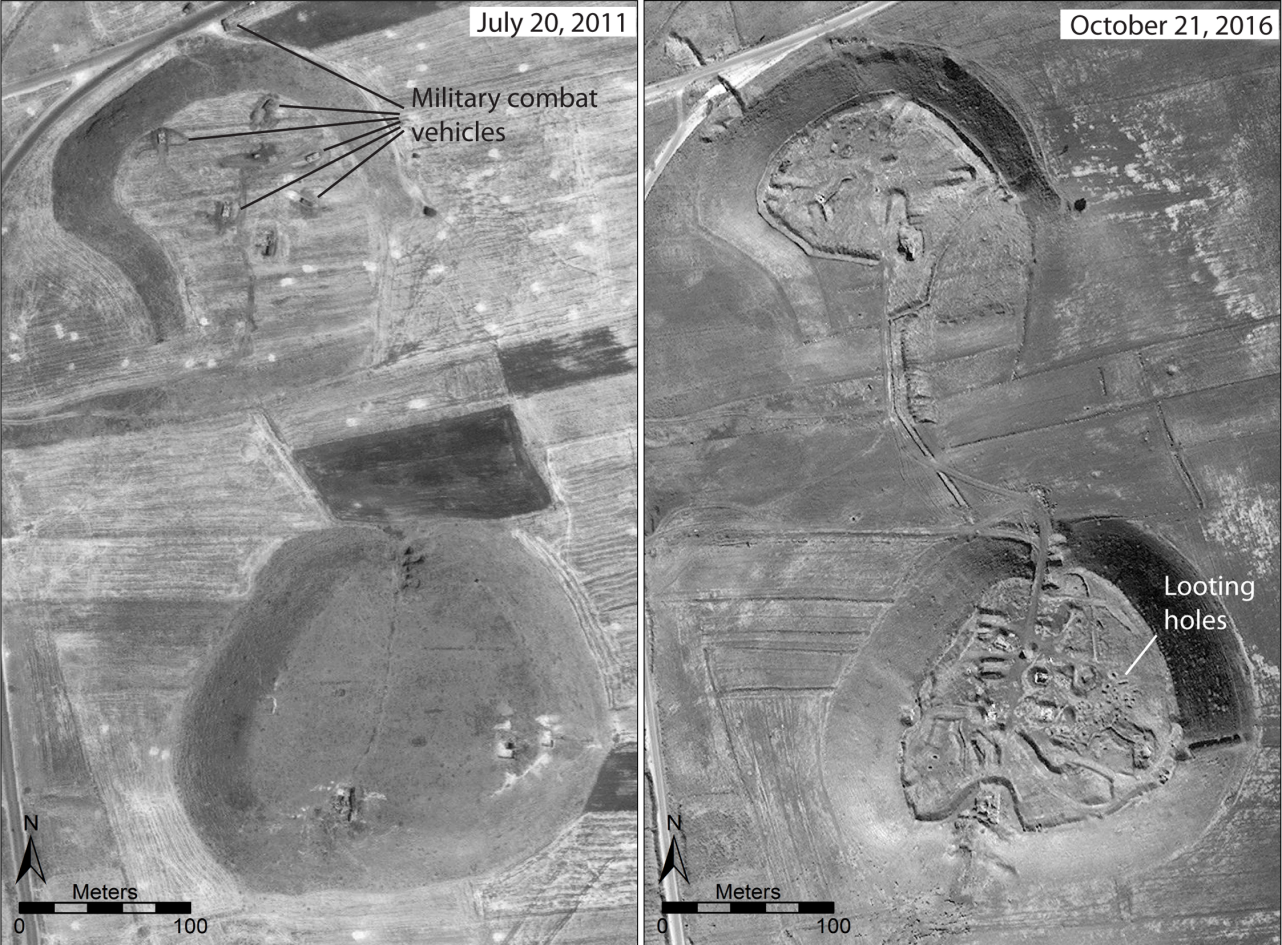
Military damage at Tell Qarqur, northwest Syria. An initial phase of relatively minor damage caused by military occupation of Tell Qarqur took place in summer 2011 (left, from [8]), but the site was severely damaged by further militarization combined with some looting during reoccupation in 2016 (right). Satellite imagery printed under a CC BY license, with permission from DigitalGlobe 2017.

**Fig 11:**
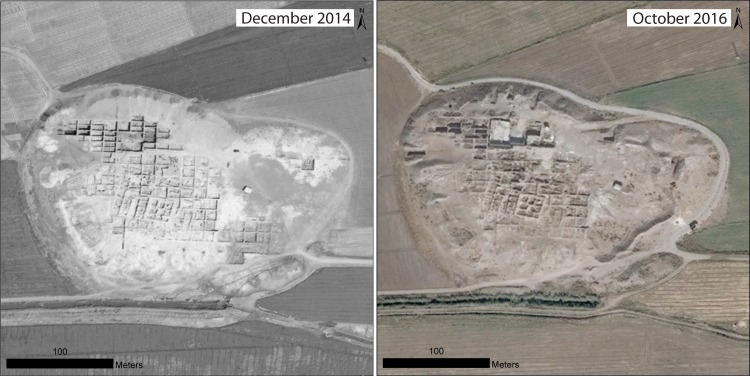
Military damage at Tell Sabi Abyad, north-central Syria. Many sites that had evaded damage during the early years of the war, such as the largely Neolithic mound of Tell Sabi Abyad, have more recently been damaged by militarization. This October 2016 image reveals the eastern side of the mound has been bulldozed. Satellite imagery printed under a CC BY license, with permission from DigitalGlobe 2017.

In many cases, sites that we previously reported as having been damaged by military activity have continued to suffer increasing levels of destruction, as in the case of the well-known Bronze Age city of Ebla in western Syria (Fig 9). Here, an artillery compound established first in 2013 [8] was expanded to six compounds combined with evidence of looting [6]. By early 2017, the site had continued to be devastated by military activity, with greatly expanded looting on the citadel around the Early Bronze Age Palace G (Fig 9A) and intense earthmoving for military purposes, as seen in the ancient city wall around the Aleppo Gate (Fig 9B). Similarly, at the site of Tell Qarqur, relatively modest damage from several military vehicle bunkers was documented during the first year of the war in 2011, but in 2016, the site was re-occupied by the Syrian military and severely damaged, while the first evidence of looting at the site became evident (Fig 10). Even at sites that had seen no damage throughout the war, such as the largely Neolithic mound of Tell Sabi Abyad in the Balikh River valley of north-central Syria, the intensification of the conflict in recent years has resulted in the construction of a military compound on the site and severe damage to the exposed archaeological excavations (Fig 11).

### New construction on sites

**Fig 12:**
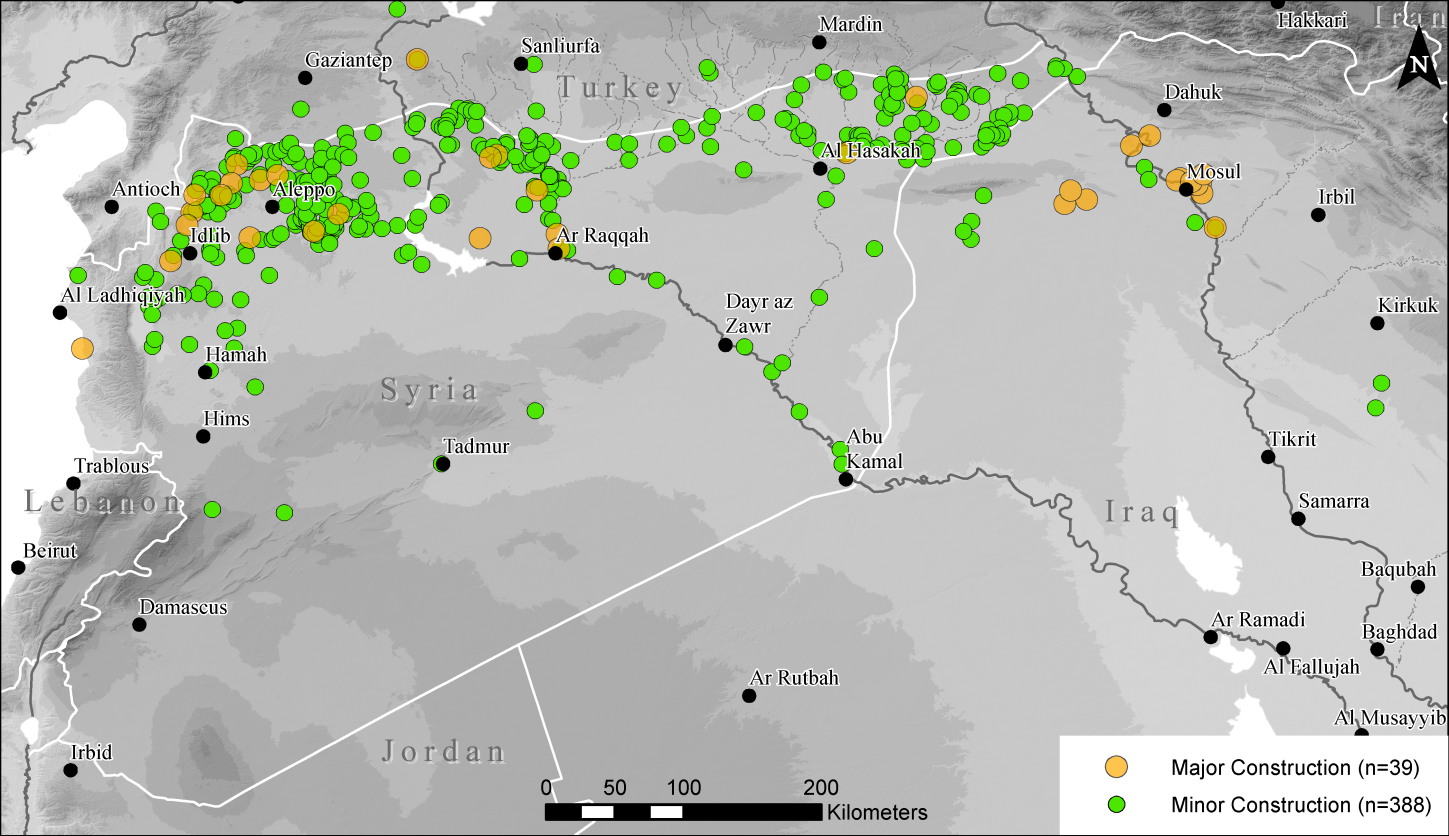
Map of sites with observed new construction since March 2011. Results show a surprising number of sites with new construction, often related to displacement of populations within Syria as well as to the rise of ISIS. Background SRTM DEM courtesy of the U.S. Geological Survey.

Our data for 2016 show a surprisingly large amount of new construction taking place on archaeological sites throughout the study area (Fig 12). In some cases, construction may be taking place opportunistically as prior to the war, expansion of residential buildings onto protected archaeological sites in Syria was restricted by the government, and like looting, may have seen some increases in the absence of civil authority. It is also demonstrably the case that in some cases new construction is a direct product of the refugee crisis. The forced displacement of unprecedented numbers of people across the entire region has resulted in the construction of vast refugee camps, and many, as for example along the Syrian-Hatay border, severely impact the major Late Roman sites known throughout the region.

**Fig 13:**
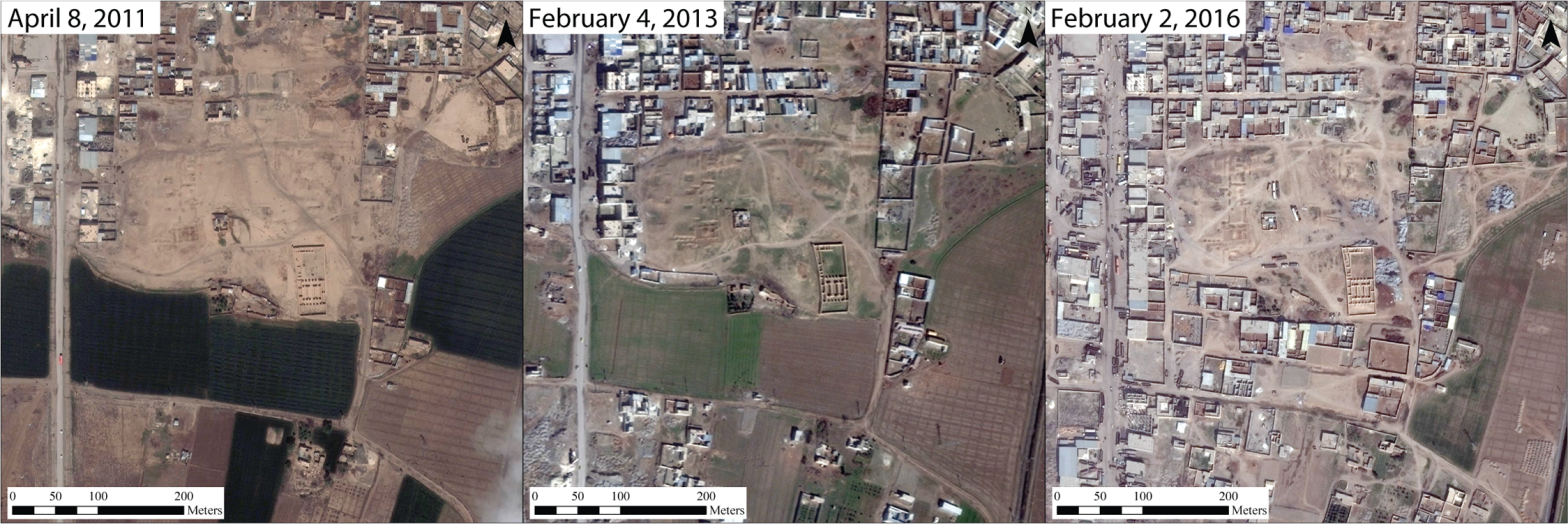
New construction in Raqqa, Syria. Urban encroachment over the ruins of the Abbasid Palace compound in Raqqa, Syria, at center of image. New construction is seen from 2013–2016 during ISIS occupation of the city. Satellite imagery printed under a CC BY license, with permission from DigitalGlobe 2017.

In other cases, construction is more directly linked to shifting political regimes. In Raqqa, Syria, we see a large amount of new construction activity that has destroyed some of the few remaining areas of the Late Roman and medieval ruins within the city (Fig 13). In the area of the former Abbasid Palace compound for example, just southeast of the modern city center, a major construction project dedicated to building what appear to be large houses was begun in 2012. A sequence of satellite images show that construction has continued at least through July 2016, with large parts of the archaeological site now completely covered by modern buildings. These construction activities, located within the *de facto* ISIS capital, are almost certainly related to ISIS activities, or at least undertaken with their approval.

**Fig 14:**
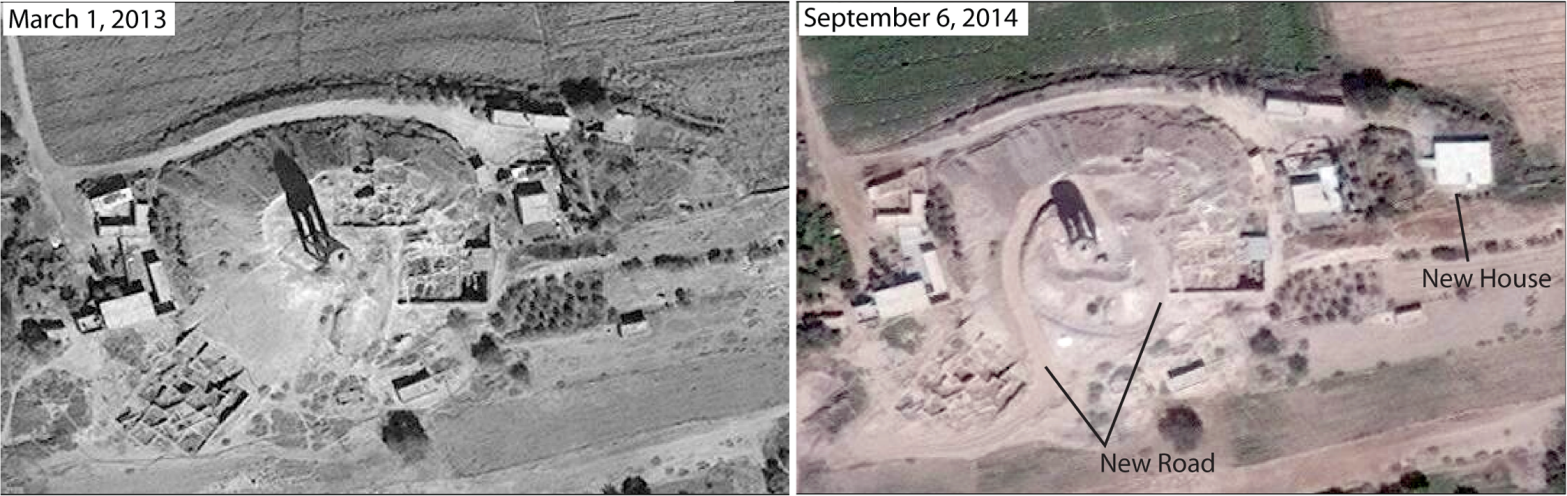
New construction at Tell Shiyukh Tahtani on the upper Euphrates River. Construction of a new road and house compound on the archaeological site of Tell Shiyukh Tahtani took place during ISIS military occupation. Satellite imagery printed under a CC BY license, with permission from DigitalGlobe 2017.

We also document cases in which construction may be linked more directly to ISIS military activities. For example, at the site of Tell Shiyukh Tahtani on the east bank of the Euphrates River in northern Syria, imagery reveals construction of a new large house compound on the eastern side of the mound between February and September 2014 (Fig 14). House construction is contemporary with the top of the site being bulldozed for construction of a new road, which has leveled several excavation areas. The site was home to an Italian-led excavation for many years [50,51], and the excavators who are still in communication with members explained that the site had been occupied by ISIS, who had militarized the top of the mound (where a large water tower is located). The eastern side of the mound was owned by a family that has reportedly fled the village, suggesting that the new construction was undertaken by ISIS in support of their military occupation of the mound.

### Atypical forms of site damage

**Fig 15:**
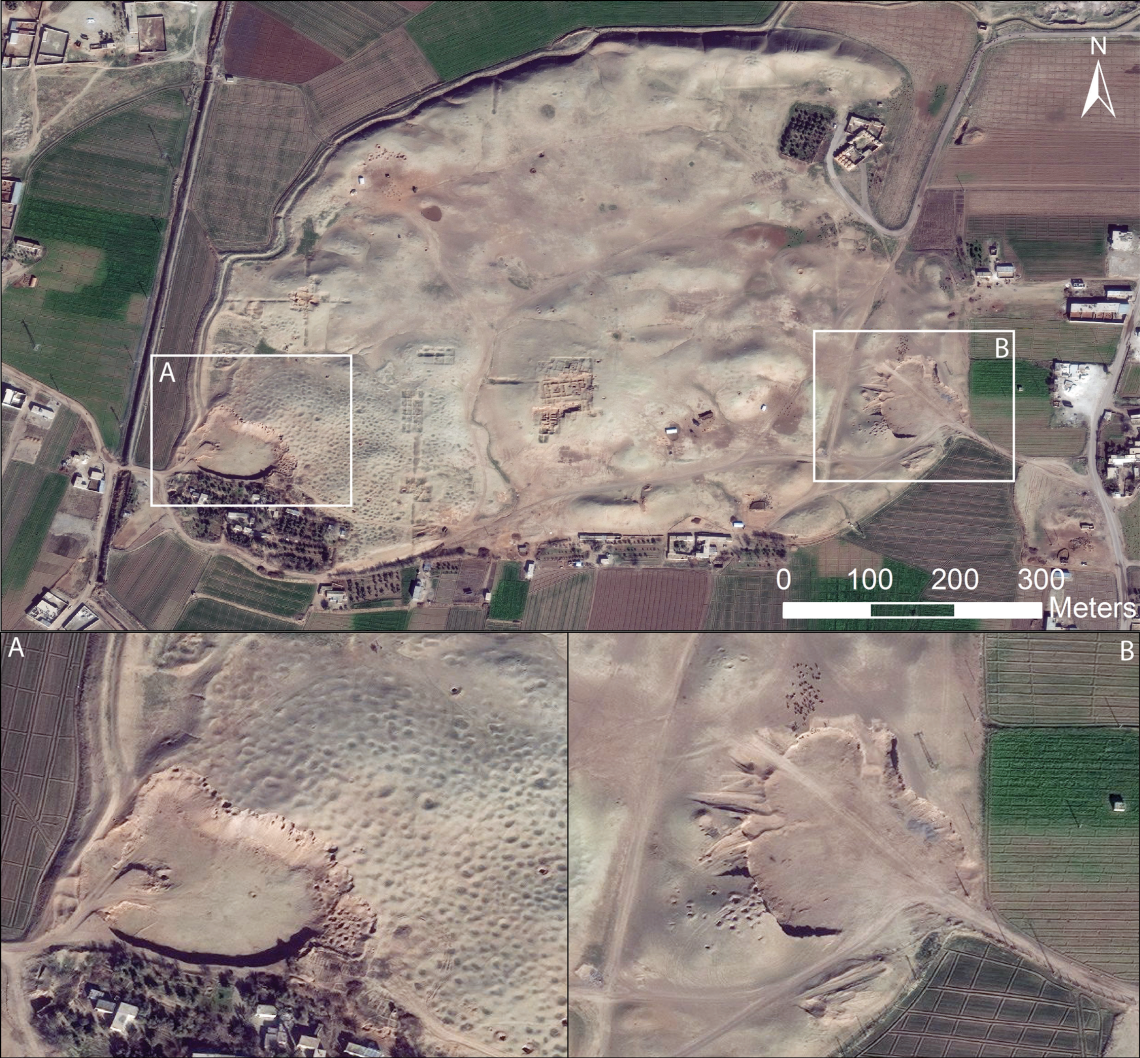
Looting and earthmoving at Tell Bi’a, Raqqa, Syria. While the southeastern Roman and medieval component of the largely Bronze Age mound of Tell Bi’a has a long history of looting, there was little evident damage to the site during the early years of the war. However, imagery reveals renewed looting and wholescale removal of several hectares of the site in 2015. Satellite imagery printed under a CC BY license, with permission from DigitalGlobe 2017.

Our research has occasionally documented forms of damage to sites that do not fit neatly into any of the major categories of damage we record. For example, the site of Tell Bi’a, just outside modern Raqqa, imagery reveals a massive earthmoving effort in which several square hectares of the major mound have been removed *en masse* (Fig 15). The site is best known for its Bronze Age palatial architecture, but a long history of looting focused largely on the later Roman and medieval remains on the southwestern corner of the site. In 2015, new looting holes appeared on these parts of the site for the first time since the war began, and soon thereafter these portions of the mound were simply removed. The purpose of the removal, whether for retrieval of antiquities off site or perhaps simply for use as construction fill, remains difficult to determine, but our analysis has identified numerous other examples of large scale earth removal from sites, all in ISIS-held areas.

**Fig 16:**
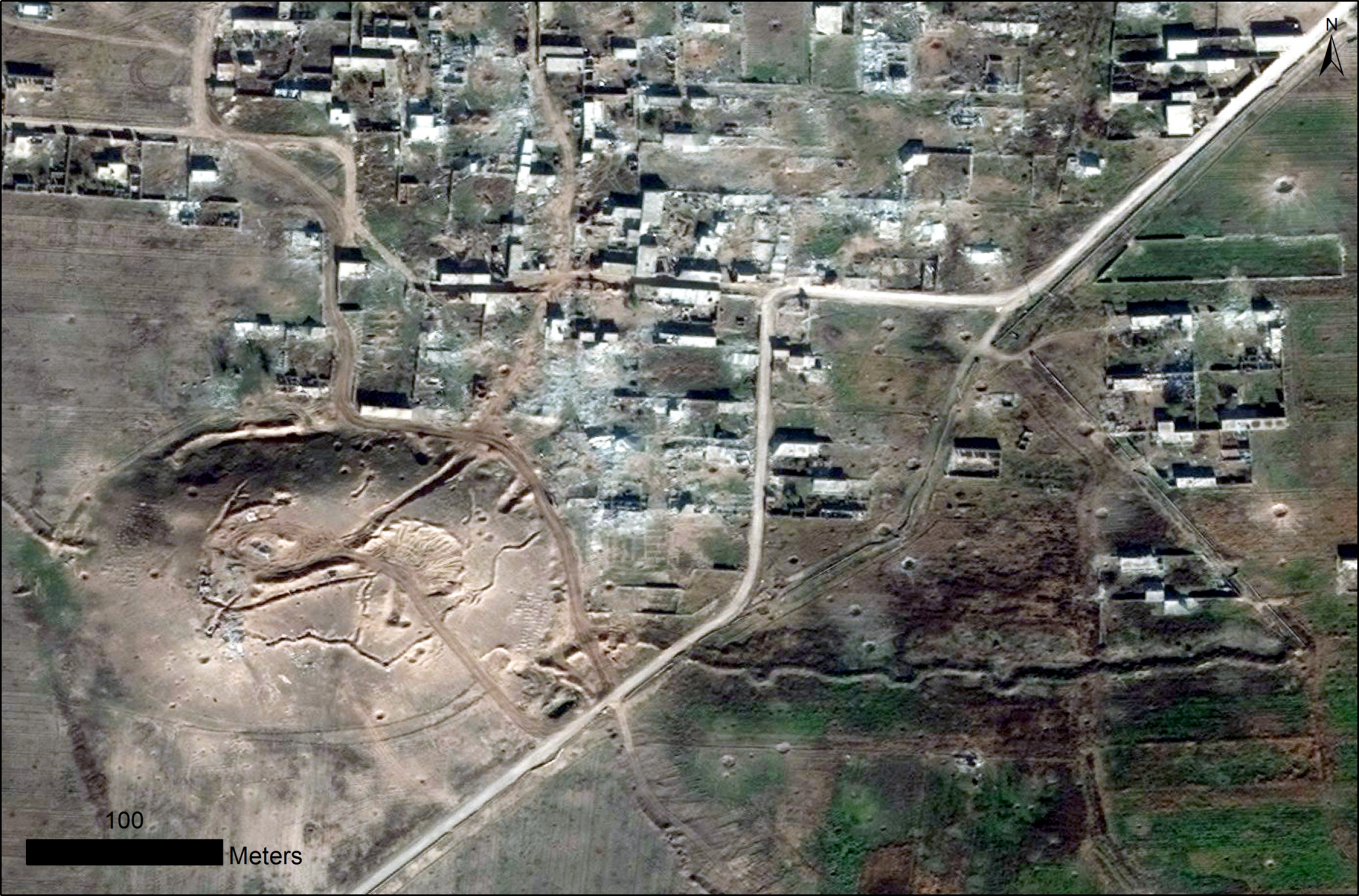
Tell Na’am in the Jabbul Plain of central Syria. Following a history of military trenching and looting dating back to 2013, in 2015 the site and adjacent village suffered heavy bombardment. Satellite imagery printed under a CC BY license, with permission from DigitalGlobe 2017.

We have often also documented direct damage to sites by ordinance, typically in the most heavily contested parts of northern Syria and Iraq, where bomb craters are evident on top of archaeological sites, often in association with military trenches that were previously dug into them, as in the case of Tell Na’am, a small mound in the Jabbul Plain of central Syria that sites at the edge of a modern village by the same name (Fig 16). In this case, earthmoving at the site was attested in mid-2013, with the first full-scale defensive earthworks constructed on the mound by August 2014 associated with moderate looting of the site. An image from December 2015 then illustrates the results of intense artillery or aerial bombardment, with dozens of craters across the site and many of the houses destroyed (Fig 16). In a similarly sad note, we also observe frequent instances throughout Syria in which modern cemeteries, often placed on top of archaeological sites, have seen rapid expansion in recent years. These sorts of observations are stark reminders of the unfolding human tragedy that is documented alongside archaeological site damage in our dataset and observations.

## Discussion

Results presented above seek to illustrate some of the myriad ways in which a large database of observations made via satellite imagery-based analyses can reveal patterns in the geographic distribution, timing, severity, and types of damage to archaeological sites in conflict zones. Analysis shows that looting has been widespread since the start of the war throughout all parts of Syria, with the worst-affected areas experiencing an order-of-magnitude-scale increase compared to the years prior to the war. Prominent mounded sites and more well-known sites are much more frequently targeted by looters, and we also can document a preference for sites of the Roman and medieval periods, and to a lesser extent sites of the late third and early second millennium BC. However, our data also show that the overall rate of looting in Syria has been gradually decreasing since late 2014, possibly as a result of depopulation due to refugee emigration, extreme violence in some areas, or the saturation of the local antiquities market. The decline in looting rates correspond with the commencement of U.S.-led coalition air strikes in August 2014 [52,53], a peak in the overall number of internally displaced persons [54–56], as well as the imposition of punishments against unsanctioned looting in ISIS-controlled areas [57]. In contrast, looting in neighboring parts of southern Turkey and northern Iraq was comparatively rare prior to the war, and appears negligible since 2012. The striking difference in looting rates and severity across national borders, even within areas currently held by ISIS in eastern Syria and northern Iraq, show the degree to which looting likely remains in large part a local phenomenon, driven by longstanding practices and notions about what sites are likely to yield valuable finds and how to access these materials.

While the picture of looting is complex, damage to archaeological sites from earthmoving, construction, and, in particular, militarization has been steadily increasing over the course of the war. Despite much scholarly and media attention to military damage at well-known sites such as Apamea, Ebla, and Tell Qarqur, conversion of these ancient cities into military compounds has only intensified in recent years. Continuous conflict along the Turkish-Syrian border as well as on the ISIS-Kurdish front lines in northern Iraq have similarly seen widespread militarization of sites since 2014 by all parties to the conflict. Combined with unfettered construction activities due to the massive movement of displaced refugees, as well as by the shifting political control of key cities, we expect that earthmoving and construction-related damage may prove to be even more devastating to archaeological and heritage sites than looting.

Our findings regarding the war in Syria illustrate the power of our methodology, which employs careful, methodical assessment of sites by trained analysts, producing a broad suite of observations that are replicable and queryable across a range of parameters. Critically, our project began by carefully considering the questions we sought to address, including where and when damage was occurring, the types of sites most at risk, and how various forms of damage were correlated with each other or with political, military, or other factors. We then structured our database to log observations in a manner that would facilitate a wide range of spatio-temporal queries designed to answer these questions. Other outwardly similar efforts have not employed this rigorous approach to recording damage, and thus are less able to produce meaningful insights. For example, our colleagues working on other aspects of the large ASOR CHI project have done exemplary research bringing together ground-based observations of site damage with careful imagery-based analyses to reveal details regarding the situation at key sites including Nimrud, Palmyra, and Mosul [5]. However, the team also sought to quickly triage damage at more than 6000 other sites, recording whether sites had “no visible, minor, some, or major” damage (see Fig 1 in [5]). This approach enables quick identification of sites with severe conflict-related damage, but because all forms of damage ranging from looting, to construction, to submersion behind a reservoir are conflated into a single category—“damage”—resultant data does little to elucidate the causes, timing, or nature of damage.

While we believe that our results are a good illustration of the effectiveness of our methods, our approach runs counter to some recent archaeological remote sensing trends that favor either automation or crowd-sourcing of observations. For example, numerous studies in recent years have sought to develop automated, machine-learning approaches to identification of archaeological features in remote sensing datasets (e.g., [58–61]), with variable degrees of success [25]. The most successful efforts have been those that combine spectral and object or shape-based attributes to locate features of regular size, shape, and reflectance such as circular, stone built tombs in arid regions of upland Yemen (e.g., [59]). In principle, looting holes could be recognized through similar methods, and Lauricella et al. [38] are successful in automating identification of looting holes, within a margin of error, in a case study in Afghanistan using full-spectrum GeoEye satellite imagery. Similarly, Tapete et al. [41] utilize a high-resolution satellite radar dataset from the TerraSAR-X program to look for textural and reflectance changes after sites are looted, focusing on the well-known case of Apamea in western Syria. Bowen et al. [62] similarly develop a sophisticated algorithm to identify looting pits in a single pan-sharpened image around a pyramid field in Upper Egypt, with good results for this small area, although how well the method would work in more diverse landscapes across multiple images remains unknown. Critically, as with all automated methods, these varied approaches are good at recognizing regularly-sized looting holes, but are much less able to identify other forms of damage visible in satellite imagery, such as earthmoving, construction, agricultural intensification, militarization, or ordinance damage. Indeed, many of the most insightful observations we have made come from the careful, textured analysis we undertake, something that cannot be automated. Nonetheless, as machine learning methods improve, it is likely that such approaches will form an increasingly valuable complement to the analyst-driven methods we employ.

Some scholars have alternatively argued for the possibilities of crowd-sourcing in archaeology, seeking to leverage the enthusiasm of a vast, anonymous (and unpaid) network of analysts. While projects that focus the crowd’s efforts on relatively rote tasks in which there is a built-in check on quality have seen some success (e.g., [63]), those that rely on a crowd to undertake primary analysis of remote sensing datasets are more problematic. In our own experiment, teams of Dartmouth students enrolled in a class on cultural heritage issues received a lecture and reading on our project, and then attempted to replicate our results. With each of six teams examining datasets of around 300 sites each that were known to include 15–20% looted sites, all teams failed to identify 90–100% of known looting incidents, but incorrectly identified looting at many other sites, commonly mistaking orchards, haystacks, or cemeteries for looting. These abysmal results are mirrored by the pilot study for the TerraWatchers project, an effort which aims to use observations by volunteers to monitor looting in the Middle East, and which found that student participants were only able to correctly identify 7% of looted sites, leading project director Stephen Savage to comment, “in the initial mission, the false positives ended up creating much more work for me than if I had done all the analysis myself” [64]. With additional training and resources, the TerraWatchers team was able to improve results to 39% accuracy [5,22], but there remains no way for researchers to know which observations are correct without independently verifying all positive and negative assessments. Parcak’s highly-publicized, TED-funded GlobalXplorer project [65] seeks to involve a much larger group of volunteers, with the idea that multiple observations by many people will produce better results, although in this case even fewer of the analysts will have had any training [23], and none of these projects have yet to publish results of their efforts. Archaeologists do not crowd-source most analytic activities, such as ceramic, lithic, or faunal analysis, because we understand that to generate meaningful data, basic observations must be made by experts who have had the necessary training and experience, and the same is true for remote sensing. Moreover, with our observations regarding archaeological site damage being the basis for real-world policy decisions, it is imperative that we ensure that our data are as accurate as possible, and to be clear about areas of uncertainty.

Finally, a number of scholars have recently questioned the ethics of undertaking imagery-based analysis of archaeological site damage in wartime [10], particularly as the practice has rapidly proliferated in popular media and non-specialist publications (e.g., [7,9,66]). Others question whether employment of satellite imagery towards monitoring archaeological sites in conflict zones might represent, along with other emerging practices, a form of digital colonialism [67]. While some of these concerns have merit, the fact remains that the imagery resources we rely upon exist in the public domain, and can be acquired by any media organization, government, or interest group; indeed they can be purchased by anyone with a valid credit card. Thus, it remains our view that offering informed, dispassionate, data-driven analysis is a critical function that can only be achieved by projects like ours, and that an absence of expert input will only lead to the proliferation of misinformation, unfortunate misunderstandings, or outright manipulation of data to achieve political ends.

## Conclusions

Research reported herein has sought to develop methodologies for effective and efficient monitoring of damage to archaeological and heritage sites using satellite imagery, and to deploy these approaches in building a better understanding of regional patterns in damage occurring in the context of the Syrian Civil War. Our results offer a unique perspective on looting and other forms of site damage, complementary to but distinct from observations and analyses based on either direct reporting or site-specific studies. We are able to monitor damage at thousands of sites in remote and dangerous regions, and by doing so we are also able to produce detailed statistics on the rate, severity, timing, and location of damage that would otherwise be impossible to assess accurately. Results therefore offer valuable information for mitigation efforts, reconstruction planning, and illicit antiquities trafficking enforcement, as well as demonstrating more broadly the power of this emerging approach as a cultural heritage management tool.

There are many specific ways in which our results may be of use to antiquities officials, heritage professionals, and archaeologists in reconstruction or future damage mitigation efforts whenever the security situation in Syria and northern Iraq makes this feasible. For example, since ISIS forces were driven out of parts of Northern Iraq earlier this year, Kurdish and Iraqi antiquities authorities have already begun assessment and restoration efforts, in cooperation with UNESCO and other international organizations, at the iconic sites of Nineveh (in Mosul), Nimrud, and elsewhere [68,69]. The efforts of organizations like the Iraqi Institute for Conservation of Antiquities and Heritage in Erbil, which seeks to train local antiquities and conservation professionals as well as provide assistance in cultural heritage management issues, can be significantly aided by detailed knowledge of the scope and severity of site damage in the region. As more areas of the war zone return to less violent and volatile conditions in the future, similar damage assessment efforts will hopefully be undertaken, and our detailed dataset will help in planning how to allocate resources and personnel.

With reporting that ISIS was profiting from the sale of antiquities looted during the war, there has been significant interest in the illicit antiquities market by governments, media, and the public. Remote sensing-based observations do not enable us to draw a direct line between particular objects and looted sites. However, our results do illustrate the types of sites most heavily targeted by looters, and thus suggest the sorts of artifacts of which enforcement agencies, antiquities dealers, and prospective buyers should be most wary. In the course of the past several years, the most severe looting has generally been focused on sites of the Roman and late Roman period (such as Apamea, Palmyra, and Dura Europos), where looters find abundant mosaics, mortuary statuary, glass and coins, as well as on sites of the Early and Middle Bronze Age (such as Ebla, Mari, and Tell Bi’a), where looters likely recover cuneiform tablets, cult statuary, bronze objects, and jewelry. Beyond specific observations of the type of artifacts likely to be appearing on the antiquities market, our findings also highlight the close relationship between looting activities and the larger humanitarian crisis in Syria and surrounding regions. The most intense looting appears consistently in areas occupied by military forces and where conflict has been pervasive. The fact that the sale of looted antiquities is not only contributing to potential funding of terrorist organizations like ISIS, but also can be seen as a direct product of the war itself and the terrible suffering it has brought, should add urgency to international efforts to curb the illicit antiquities trade and provide context to media reports and public conversation on the broader issue of the antiquities market.

As we consider potential applications of our methodologies in the future, a key issue concerns the lack of comprehensive archaeological and heritage site inventories in many parts of the world. Our project began as a response to a crisis situation, and thus was largely reactive, pulling together existing resources to achieve results quickly. As no comprehensive archaeological and heritage site database existed for Syria, we cobbled together a functional database largely derived from our earlier research project, but it remains imperfect in many respects because it was not designed to be a cultural heritage management inventory. In the year prior to the start of our project, the International Committee for the Blue Shield, an organization dedicated to preserving and protecting heritage sites during times of conflict and crisis, had similarly scrambled to assemble a “Do-Not-Strike” list when the US military was making preparations for possible airstrikes in Syria under President Obama in 2013. A key lesson from the war in Syria should be that archaeologists, government agencies, and international heritage organizations should devote more resources to development of comprehensive archaeological and heritage site databases *before* conflict or crisis occurs. In Syria, an international consortium of archaeologists known as SHIRIN [70] is now working to develop a comprehensive site inventory for use in postwar reconstruction; the University of Chicago’s Oriental Institute has developed a similar database alongside robust management infrastructure in Afghanistan [71]; and the ASOR Cultural Heritage Initiatives is now working to create site inventories and management capacities in Libya. Much work remains to be done in these and other countries around the world, but the development of archaeological and heritage inventories will enable future management and monitoring efforts to not simply be undertaken as a response to crisis, but rather as part of a more comprehensive cultural heritage management strategy.

Finally, our results help to demonstrate the power of high-resolution satellite imagery, if collected frequently and recently, to revolutionize cultural heritage monitoring efforts. Yet the potential of this approach remains largely unrealized in most parts of the world, being hamstrung by the barriers to imagery access erected by private satellite imaging companies. Purchasing current, high-resolution satellite imagery remains exorbitantly expensive, particularly for longitudinal, large-scale monitoring efforts like ours which require numerous images for thousands of locations. Our research has only been possible through a partnership with the US Department of State, which provided our team with free access to the remarkable resource that DigitalGlobe-collected imagery represents. Currently however, few researchers or heritage professionals can get similar access, problematizing replication of our work in Syria or applying similar methods elsewhere in the world. Improved access to high-resolution satellite imagery, which is already paid for almost entirely by public funds in both the United States and Europe, would enable not only heritage monitoring efforts, but a whole range of other critical activities including monitoring the effects of natural disasters, such as the recent flooding in Houston, Texas, following Hurricane Harvey (e.g., [72]), human rights catastrophes like that unfolding currently in Myanmar (e.g., [73]), as well as longer-term research in fields as diverse as ecological sciences, urban planning, and public health. The public benefits of improved imagery access are enormous, and results of our work should add fuel to efforts to make satellite imagery purchased with public funds available for the public good.

## References

[1] Daniels B, Hanson K. Archaeological Site Looting in Syria and Iraq: A Review of the Evidence In: Desmarais F, editor. Countering illicit traffic in cultural goods the global challenge of protecting the world’s heritage. Paris: The International Council of Museums; 2015 pp. 83–94.

[2] Al Quntar S, Hanson K, Daniels BI, Wegener C. Responding to a Cultural Heritage Crisis: The Example of the Safeguarding the Heritage of Syria and Iraq Project. Near Eastern Archaeology. 2015;78: 154–160. doi: 10.5615/neareastarch.78.3.0154

[3] Harmanşah Ö. ISIS, Heritage, and the Spectacles of Destruction in the Global Media. Near Eastern Archaeology. 2015;78: 170–177. doi: 10.5615/neareastarch.78.3.0170

[4] Danti MD. Ground-Based Observations of Cultural Heritage Incidents in Syria and Iraq. Near Eastern Archaeology. 2015;78: 132–141.

[5] Danti M, Branting S, Penacho S. The American Schools of Oriental Research Cultural Heritage Initiatives: Monitoring Cultural Heritage in Syria and Northern Iraq by Geospatial Imagery. Geosciences. 2017;7: 95 doi: 10.3390/geosciences7040095

[6] Casana J. Satellite Imagery-Based Analysis of Archaeological Looting in Syria. Near Eastern Archaeology. 2015;78: 142–152.

[7] Savage SH. Satellite Images Don’t Lie: What it’s like to be an archaeologist watching ISIS and other groups destroy important sites in the Middle East. Slate. 31 Mar 2015. Available: http://www.slate.com/articles/technology/future_tense/2015/03/satellite_images_show_isis_other_groups_destroying_archaeological_sites.html. Accessed 6 Oct 2017.

[8] Casana J, Panahipour M. Satellite-Based Monitoring of Looting and Damage to Archaeological Sites in Syria. Journal of Eastern Mediterranean Archaeology & Heritage Studies. 2014;2: 128–151. doi: 10.5325/jeasmedarcherstu.2.2.0128

[9] Bjørgo E, Boccardi G, Cunliffe E, Fiol M, Jellison T, Pederson W, et al. CHS-Syria: Satellite-based Damage Assessment to Cultural Heritage Sites in Syria. In: UNITAR [Internet]. 10 Dec 2014 [cited 14 Nov 2017]. Available: http://www.unitar.org/unosat/chs-syria

[10] Wolfinbarger S, Drake J, Ashcroft E, Hanson K. Ancient History, Modern Destruction: Assessing the Current Status of Syria’s World Heritage Sites Using High-Resolution Satellite Imagery. In: American Association for the Advancement of Science (AAAS) [Internet]. 16 Sep 2014 [cited 16 Oct 2017]. Available: https://www.aaas.org/page/ancient-history-modern-destruction-assessing-current-status-syria-s-world-heritage-sites-using

[11] Cunliffe E, Durham University, Global Heritage Fund. Damage to the soul: Syria’s cultural heritage in conflict [Internet]. Palo Alto, Calif.: Global Heritage Fund; 2012 Available: http://ghn.globalheritagefund.com/uploads/documents/document_2107.pdf

[12] Cunliffe EL. Satellites and site destruction: an analysis of modern impacts on the archaeological resource of the ancient Near East. [Internet]. Ph.D. Dissertation, Durham University. 2013. Available: http://etheses.dur.ac.uk/8472/

[13] Danti M, Branting S, Paulette T, Cuneo A. Report on the Destruction of the Northwest Palace at Nimrud. In: ASOR Cultural Heritage Initiatives [Internet]. 5 May 2015 [cited 15 Nov 2017]. Available: http://www.asor-syrianheritage.org/report-on-the-destruction-of-the-northwest-palace-at-nimrud/

[14] Cuneo A, Penacho S, Barnes Gordon L. Special Report: Update on the Situation in Palmyra. In: ASOR Cultural Heritage Initiatives [Internet]. 3 Sep 2015 [cited 15 Nov 2017]. Available: http://www.asor-syrianheritage.org/special-report-update-on-the-situation-in-palmyra/

[15] Stone EC. Archaeological Site Looting: The Destruction of Cultural Heritage in Southern Iraq In: Emberling G, Hanson K, editors. Catastrophe! The Looting and Destruction of Iraq’s Past. Chicago: The Oriental Institute of the University of Chicago; 2008 pp. 65–80.

[16] Stone EC. Patterns of looting in southern Iraq. Antiquity. 2008;82: 125–138. doi: 10.1017/S0003598X00096496

[17] Stone EC. An Update on the Looting of Archaeological Sites in Iraq. Near Eastern Archaeology. 2015;78: 178–186. doi: 10.5615/neareastarch.78.3.0178

[18] Contreras DA, Brodie N. Shining Light on Looting: Using Google Earth to Quantify Damage and Raise Public Awareness. SAA Archaeological Record. 2010;10: 30–33.

[19] Contreras DA, Brodie N. The Utility of Publicly-Available Satellite Imagery for Investigating Looting of Archaeological Sites in Jordan. Journal of Field Archaeology. 2010;35: 101–114. doi: 10.1179/009346910X12707320296838

[20] Brodie N, Contreras DA. The Economics of the Looted Archaeological Site of Bab Edh-Dhra’: A View from Google Earth In: Lazrus PK, Barker AW, editors. All the king’s horses: essays on the impact of looting and the illicit antiquities trade on our knowledge of the past. Washington, D.C.: Society for American Archaeology Press; 2012 pp. 9–24.

[21] Rayne L, Bewley R. Using satellite imagery to record endangered archaeology. Remote Sensing and Photogrammetry Society Archaeology Special Interest Group (RSPSoc Archaeology SIG) Newsletter. 2016; 15–20.

[22] Savage SH, Johnson A, Levy TE. TerraWatchers, Crowdsourcing, and At-Risk World Heritage in the Middle East. Heritage and Archaeology in the DigitalAge. Springer, Cham; 2017 pp. 67–77. doi: 10.1007/978-3-319-65370-9_4

[23] Parcak S, Mumford G, Childs C. Using Open Access Satellite Data Alongside Ground Based Remote Sensing: An Assessment, with Case Studies from Egypt’s Delta. Geosciences. 2017;7: 94 doi: 10.3390/geosciences7040094

[24] Afghan Heritage Mapping Partnership (AHMP). In: The Oriental Institute of the University of Chicago [Internet]. [cited 16 Nov 2017]. Available: https://oi.uchicago.edu/research/camel/afghan-heritage-mapping-partnership

[25] Casana J. Regional-Scale Archaeological Remote Sensing in the Age of Big Data. Advances in Archaeological Practice: A Journal of the Society for American Archaeology. 2014;2: 222–233.

[26] Casana J. Beyond Survey Boundaries: Satellite Remote Sensing-based Classification and Dating of Archaeological Sites in the Northern Fertile Crescent In: Lawrence D, Altaweel M, Philip G, editors. New Agendas in Remote Sensing and Landscape Archaeology in the Near East. University of Chicago Press; 2017.

[27] Casana J, Cothren J. The CORONA Atlas Project: Orthorectification of CORONA Satellite Imagery and Regional-Scale Archaeological Exploration in the Near East In: Comer D, Harrower MJ, editors. Mapping Archaeological Landscapes from Space. New York: Springer; 2013 pp. 31–41.

[28] Meyers EM, American Schools of Oriental Research. The Oxford encyclopedia of archaeology in the Near East. New York: Oxford University Press; 1997.

[29] Bagnall R, Talbert RJA, Bond S, Becker J, Elliott T, Gillies S, et al. Pleiades: A community-built gazetteer and graph of ancient places [Internet]. 2006 [cited 16 Nov 2017]. Available: http://pleiades.stoa.org

[30] The Digital Archaeological Atlas of the Holy Land [Internet]. [cited 16 Nov 2017]. Available: https://daahl.ucsd.edu/DAAHL/

[31] Casana J, Cothren J, Kalayci T. Swords into Ploughshares: Archaeological Applications of CORONA Satellite Imagery in the Near East. Internet Archaeology. 2012;32 Available: http://intarch.ac.uk/journal/issue32/2/toc.html

[32] Ur J. SPYING ON THE PAST: Declassified Intelligence Satellite Photographs and Near Eastern Landscapes. Near Eastern Archaeology. 2013;76: 28–36. doi: 10.5615/neareastarch.76.1.0028

[33] Akkermans PMMG, Schwartz GM. The Archaeology of Syria: from Complex Hunter-Gatherers to Early Urban Societies (c. 16,000–300 BC). New York: Cambridge University Press; 2003.

[34] Burns R. The monuments of Syria: a guide. London: I.B. Tauris; 2009.

[35] Kalayci T. Settlement Sizes and Agricultural Production Territories: A Remote Sensing Case Study for the Early Bronze Age in Upper Mesopotamia. STAR: Science & Technology of Archaeological Research. 2016;2: 217–234. doi: 10.1080/20548923.2016.1247512

[36] Parcak S. Archaeological Looting in Egypt: A Geospatial View (Case Studies from Saqqara, Lisht, and el Hibeh). Near Eastern Archaeology. 2015;78: 196–203. doi: 10.5615/neareastarch.78.3.0196

[37] Cunliffe E. Remote Assessments of Site Damage: A New Ontology. International Journal of Heritage in the Digital Era. 2014;3: 453–473. doi: 10.1260/2047-4970.3.3.453

[38] Lauricella A, Cannon J, Branting S, Hammer E. Semi-automated detection of looting in Afghanistan using multispectral imagery and principal component analysis. Antiquity. 2017;91: 1344–1355. doi: 10.15184/aqy.2017.90

[39] Hammer E. New Research Directions in the Camel Lab. Oriental Institute News & Notes. 2016Winter 2016: 4–9.

[40] Lasaponara R, Leucci G, Masini N, Persico R. Investigating archaeological looting using satellite images and GEORADAR: the experience in Lambayeque in North Peru. Journal of Archaeological Science. 2014;42: 216–230. doi: 10.1016/j.jas.2013.10.032

[41] Tapete D, Cigna F, Donoghue DNM. ‘Looting marks’ in space-borne SAR imagery: Measuring rates of archaeological looting in Apamea (Syria) with TerraSAR-X Staring Spotlight. Remote Sensing of Environment. 2016;178: 42–58. doi: 10.1016/j.rse.2016.02.055

[42] Dewar RE. Incorporating Variation in Occupation Span into Settlement-Pattern Analysis. American Antiquity. 1991;56: 604–620. doi: 10.2307/281539

[43] Keller A. Documenting ISIL’s Antiquities Trafficking: The Looting and Destruction of Iraqi and Syrian Cultural Heritage: What We Know and What Can Be Done. Transcript of remarks made at The Metropolitan Museum of Art, New York, NY. In: U.S. Department of State [Internet]. 29 Sep 2015 [cited 4 Oct 2017]. Available: https://2009-2017.state.gov/e/eb/rls/rm/2015/247610.htm

[44] Taub B. The Real Value of the ISIS Antiquities Trade. The New Yorker. 4 Dec 2015. Available: https://www.newyorker.com/news/news-desk/the-real-value-of-the-isis-antiquities-trade. Accessed 16 Nov 2017.

[45] CBS News. Relics from Syria not destroyed by ISIS sold on black market [Internet]. 9 Sep 2015 [cited 6 Oct 2017]. Available: https://www.cbsnews.com/videos/relics-from-syria-not-destroyed-by-isis-sold-on-black-market/

[46] Bruenig E. Between Hobby Lobby and ISIS: The Battle for Iraq’s Antiquities. The New Republic. 29 Oct 2015. Available: https://newrepublic.com/article/123262/between-hobby-lobby-and-isis-battle-iraqs-antiquities. Accessed 6 Oct 2017.

[47] Green E. Hobby Lobby Purchased Thousands of Ancient Artifacts Smuggled Out of Iraq. The Atlantic. 5 Jul 2017. Available: https://www.theatlantic.com/politics/archive/2017/07/hobby-lobby-smuggled-thousands-of-ancient-artifacts-out-of-iraq/532743/. Accessed 6 Oct 2017.

[48] IHS Markit Conflict Monitor. Study Shows Islamic State’s Primary Opponent in Syria Is Government Forces, IHS Markit Says | IHS Online Newsroom. In: IHS Markit [Internet]. 19 Apr 2017 [cited 6 May 2017]. Available: http://news.ihsmarkit.com/press-release/aerospace-defense-security/study-shows-islamic-states-primary-opponent-syria-governmen

[49] Lawler A. War, More Than ISIS, Is Destroying Syria’s Ancient Sites. National Geographic News. 25 Nov 2015. Available: http://news.nationalgeographic.com/2015/11/151125-isis-syria-satellite-images-looting-archaeology/. Accessed 20 Jan 2017.

[50] Falsone G. Tell Shiyukh Tahtani. In: del Olmo Lete G, Montero Fenollós J-L, editors. Archaeology of the Upper Syrian Euphrates the Tishrin dam Area: proceedings of the international symposium held at Barcelona, January 28 th - 30th 1998. Sabadell (Barcelona), Spain: AUSA; 1999. pp. 137–142.

[51] Sconzo P. Collapse or Continuity? The case of the EB-MB Transition at Tell Shiyukh Tahtani. Varia Anatolica. 2007;19: 267–310.

[52] U.S. Department of Defense. Special Report: Inherent Resolve: Strikes in Iraq and Syria. [Internet]. [cited 16 Apr 2017]. Available: https://www.defense.gov/News/Special-Reports/0814_Inherent-Resolve/

[53] The war against ‘Islamic State’ in maps and charts. BBC News. 3 Apr 2017. Available: http://www.bbc.com/news/world-middle-east-27838034. Accessed 4 Oct 2017.

[54] U.S. Department of State—Humanitarian Information Unit. Syria and Iraq Conflict Without Borders: 2016 in Review. In: Humanitarian Information Unit [Internet]. 7 Feb 2017 [cited 6 Oct 2017]. Available: https://hiu.state.gov/hiu-products/jpg/IraqSyria_YearInReview2016_Page1_2017Feb03_HIU_U1512.jpg

[55] Internal Displacement Monitoring Centre (IDMC). IDP Database: Syria [Internet]. 31 Dec 2015 [cited 16 Apr 2017]. Available: http://www.internal-displacement.org/database/country/?iso3=SYR

[56] Office for the Coordination of Humanitarian Affairs (OCHA). Syrian Arab Republic [Internet]. Mar 2017 [cited 16 Apr 2017]. Available: http://www.unocha.org/syria

[57] Shabi R. Looted in Syria–and sold in London: the British antiques shops dealing in artefacts smuggled by Isis. The Guardian. 3 Jul 2015. Available: https://www.theguardian.com/world/2015/jul/03/antiquities-looted-by-isis-end-up-in-london-shops. Accessed 16 Apr 2017.

[58] Harrower MJ, Schuetter J, McCorriston J, Goel PK, Senn MJ. Survey, Automated Detection, and Spatial Distribution Analysis of Cairn Tombs in Ancient Southern Arabia In: Comer D, Harrower MJ, editors. Mapping Archaeological Landscapes from Space. New York: Springer; 2013 pp. 259–268.

[59] Schuetter J, Goel P, McCorriston J, Park J, Senn M, Harrower M. Autodetection of ancient Arabian tombs in high-resolution satellite imagery. International Journal of Remote Sensing. 2013;34: 6611–6635. doi: 10.1080/01431161.2013.802054

[60] Menze BH, Ur JA. Mapping Patterns of Long-Term Settlement in Northern Mesopotamia at a Large Scale. PNAS. 2012;109: E778–E787. doi: 10.1073/pnas.1115472109 22431607 10.1073/pnas.1115472109 PMC3325708

[61] Trier ØD, Larsen SØ, Solberg R. Automatic detection of circular structures in high-resolution satellite images of agricultural land. Archaeol Prospect. 2009;16: 1–15. doi: 10.1002/arp.339

[62] Bowen EFW, Tofel BB, Parcak S, Granger R. Algorithmic Identification of Looted Archaeological Sites from Space. Front ICT. 2017;4 doi: 10.3389/fict.2017.00004

[63] Bonacchi C, Bevan A, Pett D, Keinan-Schoonbaert A. Crowd- and Community-Fuelled Archaeological Research. Early Results from the MicroPasts Project. Proceedings of the Conference Computer Applications and Quantitative Methods in Archaeology. 2015. pp. 279–288. Available: http://discovery.ucl.ac.uk/1486104/

[64] Ramsey D. UC San Diego Cyber-Archaeology Researchers Launch Crowdsourcing Portal to Monitor At-Risk Sites. In: UC San Diego UC San Diego News Center [Internet]. 15 Apr 2016 [cited 16 Oct 2017]. Available: http://ucsdnews.ucsd.edu/pressrelease/uc_san_diego_cyber_archaeology_researchers_launch_crowdsourcing_portal_to_m

[65] Stinson E. Sarah Parcak Is a Space Archaeologist. Soon You Will Be Too. In: WIRED [Internet]. Fabruary 2016 [cited 6 Oct 2017]. Available: https://www.wired.com/2016/02/sarah-parcak/

[66] Rodgers L. Syrian heritage destruction revealed in satellite images. BBC News. 19 Sep 2014. Available: http://www.bbc.com/news/world-middle-east-29255315. Accessed 16 Nov 2017.

[67] Duffton A, Ogden J. Decolonizing Digital Archaeology. Session Abstract for the Computer Applications and Quantitative Methods in Archaeology meeting, 2017. In: CAA 2017 Conference [Internet]. 2016 [cited 15 Nov 2016]. Available: http://2017.caaconference.org/program/sessions/

[68] Bassem W. Iraq steps up efforts to restore lost heritage at ancient Nimrud. Al-Monitor. 7 Aug 2017. Available: http://www.al-monitor.com/pulse/originals/2017/08/iraq-nimrud-archaeology-isis-reconstruction.html. Accessed 4 Oct 2017.

[69] Hammer J. The Salvation of Mosul. Smithsonian Magazine. Oct 2017. Available: http://www.smithsonianmag.com/history/salvation-mosul-180964772/. Accessed 4 Oct 2017.

[70] Shirin International | Protecting Syria Heritage [Internet]. [cited 16 Nov 2017]. Available: http://shirin-international.org/

[71] Hammer E. Center for Ancient Middle Eastern Landscapes (CAMEL). In: Stein GJ, editor. The Oriental Institute 2015–2016 Annual Report. The Oriental Institute, Chicago: University of Chicago; 2016. pp. 18–27. Available: https://oi.uchicago.edu/about/annual-reports/oriental-institute-2015-2016-annual-report

[72] Griffin J. New satellite photos reveal extent of Harvey flooding in Houston. PBS NewsHour. 1 Sep 2017. Available: https://www.pbs.org/newshour/science/new-satellite-photos-reveal-extent-harvey-flooding-houston. Accessed 4 Oct 2017.

[73] Peçanha S, White J. Satellite Images Show More Than 200 Rohingya Villages Burned in Myanmar. The New York Times. 18 Sep 2017. Available: https://www.nytimes.com/interactive/2017/09/18/world/asia/rohingya-villages.html. Accessed 4 Oct 2017.

